# Electromagnetic Cellularized Patch with Wirelessly Electrical Stimulation for Promoting Neuronal Differentiation and Spinal Cord Injury Repair

**DOI:** 10.1002/advs.202307527

**Published:** 2024-06-13

**Authors:** Liang Wang, Hongbo Zhao, Min Han, Hongru Yang, Ming Lei, Wenhan Wang, Keyi Li, Yiwei Li, Yuanhua Sang, Tao Xin, Hong Liu, Jichuan Qiu

**Affiliations:** ^1^ State Key Laboratory of Crystal Materials Shandong University Jinan Shandong 250100 P. R. China; ^2^ Department of Neurosurgery The First Affiliated Hospital of Shandong First Medical University & Shandong Provincial Qianfoshan Hospital Jinan 250014 P. R. China; ^3^ Department of Neurosurgery, Shandong Provincial Qianfoshan Hospital Shandong University Jinan 250014 P. R. China; ^4^ Medical Science and Technology Innovation Center Shandong First Medical University and Shandong Academy of Medical Sciences Jinan 250117 P. R. China; ^5^ Department of Neurosurgery Jiangxi Provincial People's Hospital Nanchang Jiangxi 330006 P. R. China; ^6^ Institute for Advanced Interdisciplinary Research University of Jinan Jinan Shandong 250022 P. R. China

**Keywords:** cellularized patch, neural differentiation, neural stem cells, spinal cord injury, wirelessly electrical stimulation

## Abstract

Although stem cell therapy holds promise for the treatment of spinal cord injury (SCI), its practical applications are limited by the low degree of neural differentiation. Electrical stimulation is one of the most effective ways to promote the differentiation of stem cells into neurons, but conventional wired electrical stimulation may cause secondary injuries, inflammation, pain, and infection. Here, based on the high conductivity of graphite and the electromagnetic induction effect, graphite nanosheets with neural stem cells (NSCs) are proposed as an electromagnetic cellularized patch to generate in situ wirelessly pulsed electric signals under a rotating magnetic field for regulating neuronal differentiation of NSCs to treat SCI. The strength and frequency of the induced voltage can be controlled by adjusting the rotation speed of the magnetic field. The generated pulsed electrical signals promote the differentiation of NSCs into functional mature neurons and increase the proportion of neurons from 12.5% to 33.7%. When implanted in the subarachnoid region of the injured spinal cord, the electromagnetic cellularized patch improves the behavioral performance of the hind limbs and the repair of spinal cord tissue in SCI mice. This work opens a new avenue for remote treatment of SCI and other nervous system diseases.

## Graphical abstract

An electromagnetic cellularized patch is fabricated to generate wirelessly electrical stimulation under a changing magnetic field. The frequency and strength of the electrical signal can be tuned by varying the rotation speed of the magnetic field. The patch‐mediated wirelessly electrical stimulation promotes the differentiation of neural stem cells and the repair of injured spinal cord.

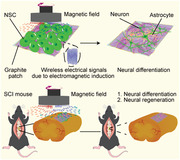

## 1 Introduction

Spinal cord injury (SCI) is a severely disabling neurological condition with symptoms of impaired mobility, pain, and autonomic dysfunction.^[^ ^1^ ^]^ The dead or damaged spinal neurons at the injury site cause the paralysis of denervated musculature and the disruption of long spinal tracts, resulting in the loss of sensation and motor control. Currently, there is no effective therapy that can cure or reverse SCI because spinal neurons are not regenerable in adults and the lesion core is often replaced by glial scars.^[^ ^2^ ^]^ Neural stem cell (NSC)‐based therapy is recognized as a promising candidate for the treatment of SCI by replacing damaged neurons to reconstruct neural circuits and restore neural function.^[^ ^3^ ^]^ It has been demonstrated that the transplantation of stem cells to damaged sites could improve the recovery of the spinal cord to a certain extent in animal models.^[^ ^4^ ^]^ However, the challenge in directing impanated stem cells to differentiate into functional neurons in the complex microenvironment of the injury site limits the therapeutic performance of stem cell‐based therapy for SCI.^[^ ^5^ ^]^ Although some neurotrophic factors and small molecule drugs are able to regulate the differentiation of NSCs,^[^ ^6^ ^]^ they face obstacles including high cost, short half‐life, and difficulty in delivery in practical applications.^[^ ^7^ ^]^

Pulsed electrical stimulation is a powerful approach for promoting the differentiation of NSCs and the repair of nerve injury.^[^ ^8^ ^]^ Electrical stimulation promotes the differentiation of NSCs by affecting membrane protein functions, such as enzyme activity, membrane receptor complexes, and ion‐transport channels. In addition, electrical stimulation plays a critical role in synaptogenesis, neuronal recruitment, and neuronal survival.^[^ ^9^ ^]^ However, conventional electrical stimulation generally involves wires and electrodes, for which implantation and removal require additional surgery. The wires, electrodes, as well as the surgery, may cause secondary injuries, inflammation, pain, and infection.^[^ ^10^ ^]^ By transforming external field energy into electrical signals, wirelessly electrical stimulation provides a new direction for the regulation of neural differentiation and injured spinal cord repair. Through this approach, additional surgery as well as the risk of infection and inflammation can be minimally avoided.^[^ ^11^ ^]^ Light or ultrasound has been explored as the external energy to apply wirelessly electrical stimulation to stem cells or neural tissues. For example, an optoelectronic material‐based scaffold could generate electrical signals under laser irradiation to promote the elongation and differentiation of neurons.^[^ ^12^ ^]^ However, the limited penetration depth of light in biological tissues restricts its use in SCI treatment, especially considering that the spinal cord is surrounded by vertebrae.^[^ ^13^ ^]^ On the other hand, piezoelectric materials have also been used to apply wirelessly electrical stimulation under ultrasound by leveraging their piezoelectric effect. For example, Chen et al. reported that a poly‐L‐lactic acid‐based piezoelectric scaffold capable of producing pulsed electrical signals under ultrasound showed outperformance in promoting neural differentiation and injured spinal cord repair.^[^ ^14^ ^]^ Unfortunately, ultrasound will attenuate sharply at the interface between different tissues or compositions. In addition, ultrasound will cause mechanical pressure as well as thermal and cavitation effects, which may cause damage to the skin and nerves.^[^ ^15^ ^]^

Electromagnetic induction was discovered by Michael Faraday in 1831 and it is a well‐known process in which a conductor placed in a changing magnetic field will produce a voltage across the conductor. By leveraging electromagnetic induction, wirelessly electrical signals can be generated on conductors under an external magnetic field, which is a noncontact and noninvasive stimulation method.^[^ ^16^ ^]^ More importantly, the magnetic field interacts weakly with biological molecules and can penetrate deep into the body without attenuation.^[^ ^17^ ^]^ To this end, the magnetic field is considered a promising external field for truly invasive and remote electrical stimulation. Although it has been demonstrated that electrical stimulation based on electromagnetic induction could influence cell behaviors,^[^ ^18^ ^]^ it has been a challenge to apply electrical stimulation to implanted stem cells and the spinal cord for SCI treatment.

**Figure 1:**
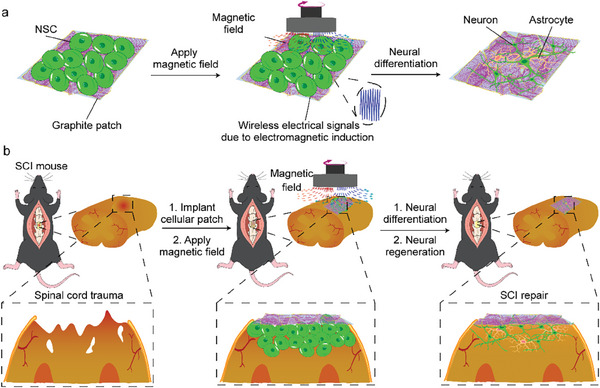
Schematic showing the electromagnetic cellularized patch capable of generating wirelessly electrical stimulation for promoting neural differentiation and SCI repair. a) Schematic showing the neural differentiation of NSCs seeded on the graphite patch under the rotating magnetic field. b) Schematic showing implantation of the cellularized patch to the subarachnoid site of the injured spinal cord can promote the repair of the injured spinal cord tissues under the rotating magnetic field.

Here, we report an electromagnetic cellularized patch that can generate wirelessly electrical stimulation under a changing magnetic field to promote neural differentiation and injured spinal cord repair (**Figure** **1**). The cellularized patch is made of slightly flaky graphite nanosheets seeded with NSCs. The graphite nanosheets were chosen because of their good biocompatibility and conductivity.^[^ ^19^ ^]^ Graphite material has been used in vivo due to its good biocompatibility.^[^ ^20^ ^]^ When a graphite patch is placed in a rotating magnetic field powered by rotating magnets, it generates a wirelessly induced voltage and induced current. The rotating magnets provide a low frequency alternating magnetic field, which does not produce as obvious an eddy current or thermal effect as a high‐frequency alternating magnetic field (100 kHz to 100 MHz).^[^ ^21^ ^]^ In addition, the strength and frequency of the electrical signal can be regulated by varying the rotation speed of the magnetic field. Electromagnetic cellularized patch‐mediated electrical stimulation promoted the neural differentiation of NSCs seeded on the patch in vitro (Figure 1a). When implanted into the subarachnoid site of the injured spinal cord of SCI mice, the cellularized patch can significantly improve the behavioral performance of the hind limbs and the tissue recovery of the injured spinal cord under the rotating magnetic field (Figure 1b). This electromagnetic cellularized patch capable of generating wirelessly electrical stimulation under a changing magnetic field has great promise in the remote and invasive treatment of SCI and other diseases of the central and peripheral nervous systems.

## 2 Results and Discussion

**Figure 2:**
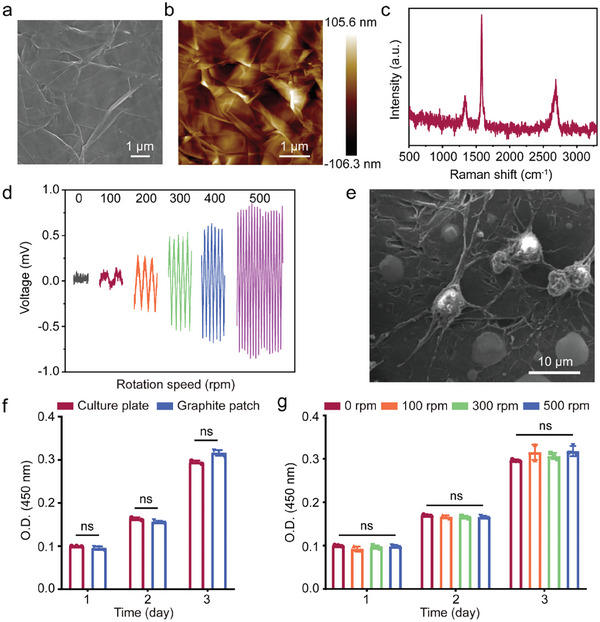
a) SEM and b) AFM images of the graphite patch. c) Raman spectra of the graphite patch. d) The voltage generated on the graphite patch when placed under a rotating magnetic field at rotation speeds of 0, 100, 200, 300, 400, and 500 rpm. e) SEM image of NSCs cultured on the graphite patch for 3 days. f) Proliferation of NSCs after culture on the graphite patch for 1, 2, and 3 days. NSCs seeded in the culture plate served as control. g) Viability of NSCs on the graphite patch when changing magnetic field at rotation speeds of 0, 100, 300, and 500 rpm was applied. The data were expressed as the mean ± standard deviation (n = 3), as analyzed by a one‐way ANOVA, with Tukey's multiple comparisons test (nsp > 0.05).

The graphite patch was made of high purity, high conductivity graphite nanosheets. **Figure** **2**a shows a scanning electron microscopy (SEM) image of the as‐fabricated graphite patch, indicating that the patch was composed of slightly flaky graphite nanosheets. The atomic force microscopy (AFM) image in Figure 2b confirms that the patch of stacked graphite nanosheets is 2–5 µm in size. The Raman spectrum in Figure 2c shows that the graphite patch exhibited 3 characteristic peaks, which are the D band at 1338 cm^−1^, the G band at 1582 cm^−1^, and the 2D band at 2688 cm^−1^. The ratio of the intensity of the G band to that of the D band was approximately 3, indicating that the graphite patch had a high degree of graphitization, as the D and G bands resulted from the vibrations of sp^2^ hybridized and sp^3^‐bonded carbon, respectively.^[^ ^22^ ^]^ In addition, the X‐ray diffraction (XRD) pattern in Figure S1 shows that the characteristic peak of graphite is obvious, indicating that the degree of graphitization is high. The high degree of graphitization of the graphite patch would provide a high conductivity, which is beneficial for the generation of wirelessly electrical signals under a changing magnetic field.

The intensity of the electrical signals generated by the graphite patch under a rotating magnetic field was assessed using an oscilloscope. A neodymium‐iron‐boron permanent magnet driven by the motor was used to provide the rotating magnetic field. The electrical signal is generated based on the electromagnetic induction effect. A 1 × 1 cm^2^ graphite patch was placed 1 cm above the magnet, where the strength of the magnetic field was 80 mT as measured by a Gaussmeter. Figure 2d shows that the induced voltages generated on the graphite patch were 0, 0.13, 0.47, 0.59, and 0.82 mV, when the magnet was rotated at 0, 100, 200, 300, 400, and 500 rpm, respectively. The periodic motion of the rotating magnetic field generated a sinusoidal pulsed electrical signal. Furthermore, the induced voltages generated on the graphite exhibited different frequencies consistent with the rotating speed. In sharp contrast, nonconductive materials, such as culture plates, did not generate voltage when exposed to the same magnetic field (Figure S2, Supporting Information). The above results demonstrate that we can maneuver the strength and frequency of wirelessly pulsed electrical signals by controlling the rotating speed of the magnetic field.

NSCs were seeded on the graphite patch to form a cellularized patch. Before seeding the cells, the graphite patch was pretreated with oxygen plasma to facilitate the cell adhesion (Figure S3, Supporting Information).^[^ ^23^ ^]^ Figure 2e shows the SEM image of the NSCs after seeding on the graphite patch for three days, demonstrating that the cells were well attached and spread on the graphite patch. The cell pseudopods were in contact with the graphite substrate, which would facilitate the electrical stimulation to work on the cells. The cell proliferation and viability on the graphite patch were evaluated by Cell Counting Kit‐8 (CCK‐8) and Live/Dead Staining Kit. The cells cultured on the culture plate served as control. The CCK‐8 results in Figure 2f show that the graphite patch exhibited good biocompatibility with the NSCs. The live/dead staining results shown in Figures S4 and S5 (Supporting Information) suggest that almost all the cells (over 95%) were alive on the graphite patch, further confirming its good biocompatibility. We further investigated whether a magnetic field or magnetic field‐induced electrical signals would influence cell activity. As shown by the CCK‐8 results in Figure 2g, changing the magnetic field at rotational speeds of 100, 300, or 500 rpm did not affect the viability of the NSCs seeded on the graphite patch. Taken together, the graphite patch had good biocompatibility with NSCs regardless of whether a changing magnetic field was applied.

**Figure 3:**
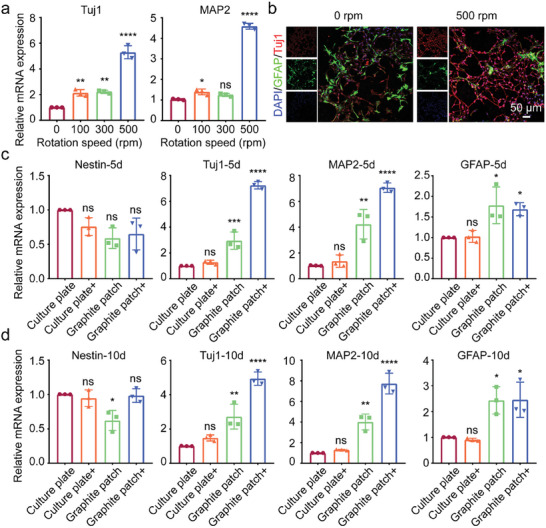
a) RT‐qPCR results of NSCs on graphite patch under magnetic field at rotation speeds of 0, 100, 300, and 500 rpm at day 5. b) Immunofluorescence staining of Tuj1 and GFAP of NSCs on graphite patch under magnetic field at rotation speed of 0 and 500 rpm at day 5. Tuj1 was stained red. GFAP was stained green. While nuclei were stained blue by DAPI. c, d) RT‐qPCR of NSCs on culture plate or graphite patch without or with (+) a 500‐rpm magnetic field at c) day 5 and d) day 10. The data were expressed as the mean ± standard deviation (n = 3), as analyzed by a one‐way ANOVA, with Tukey's multiple comparisons test (nsp>0.05, *p<0.05, **p<0.01, ***p<0.001, and ****p<0.0001 compared to the culture plate group).

To evaluate if the electrical signals generated on the graphite patch under the rotating magnetic field could promote the neural differentiation of NSCs, reverse transcription‐quantitative polymerase chain reaction (RT‐qPCR) was used to assess the expression of neuron markers of NSCs cultured on culture plate and on graphite patch under different conditions at different times. Nestin, a cytoskeletal intermediate filament initially characterized in NSCs, was selected as the marker of NSCs.^[^ ^24^ ^]^ βIII tubulin (Tuj1), a cytoskeletal protein expressed in neurons,^[^ ^25^ ^]^ and microtubule‐associated protein 2 (MAP2), a key tubulin involved in neurogenesis, microtubule assembly, and neural development were used as markers of early neurons and mature neurons, respectively.^[^ ^26^ ^]^ Glial fibrillary acid protein (GFAP), a protein mainly distributed in astrocytes of the central nervous system, is used as a marker of glial cells.^[^ ^25^ ^]^ **Figure** **3**a shows the RT‐qPCR results after NSCs on the graphite patch were applied to the magnetic field at different rotation speeds for 5 days. Compared to that of the graphite patch without changing magnetic field, the expression of Tuj1 of NSCs cultured under a magnetic field at rotation speed of 100, 300, and 500 rpm were upregulated 2.1‐, 2.2‐, and 5.3‐fold, respectively, while the expression of MAP2 were up‐regulated 1.4‐, 1.2‐, and 4.5‐fold, respectively. The RT‐qPCR results show that the changing magnetic field at 500 rpm could significantly promote the neural differentiation of NSCs on the graphite patch while the low magnetic field rotation speed could only slightly promote the neural differentiation of NSCs. This was probably because the 500‐ rpm magnetic field could induce a higher voltage on the graphite patch and thus strongly stimulate to the NSCs. Figure 3b shows the immunofluorescence staining results of NSCs on the graphite patch, in which Tuj1, GFAP, and the nuclei were stained red, green, and blue, respectively. Compared to NSCs without changing magnetic field, NSCs cultured under a 500‐ rpm rotating magnetic field exhibited a typical neuronal morphology and expressed more Tuj1. The above results demonstrated that the graphite patch‐mediated wirelessly electrical stimulation could significantly promote the differentiation of NSCs into neurons.

We then systematically investigated the influence of graphite‐mediated wirelessly electrical stimulation on neural differentiation of NSCs by setting the magnetic field rotating speed at 500 rpm. NSCs cultured on the culture plate without magnetic field (culture plate) or with a 500‐rpm magnetic field (culture plate+), and NSCs cultured on the graphite patch without magnetic field (graphite patch) or with the 500‐rpm magnetic field (graphite patch+) were subjected to the RT‐qPCR. Figure 3c,d show the RT‐qPCR results of NSCs at day 5 and day 10, respectively. There was nearly no difference in the expression of Nestin of NSCs among the groups, indicating that the graphite patch and/or the magnetic field did not alter the stemness of NSCs. In addition, regardless of whether magnetic field was applied, the expression of Tuj1, MAP2, and GFAP of NSCs in the culture plate did not change significantly, indicating that the rotating magnetic field itself has no influence on the neural differentiation of NSCs. The expression of Tuj1 of NSCs on the graphite patch was upregulated 2.94‐fold at day 5 and 2.71‐fold at day 10 compared to that of the cells on the culture plate. The expression of MAP2 on the graphite patch was upregulated 4.22‐fold at day 5 and 4‐fold at day 10. These results indicate that the graphite patch themselves slightly promoted the neural differentiation of NSCs, which was consistent with previous reports and should be attributed to the good conductivity of the graphite. When the 500‐rpm rotating magnetic field was applied to the NSCs on the graphite patch, the expression of Tuj1 was upregulated 7.2‐fold at day 5 and 4.9‐fold at day 10 while the expression of MAP2 was upregulated 7.1‐fold at day 5 and 7.6‐fold at day 10, compared to that in the culture plate group. These results demonstrated that the graphite patch‐mediated wirelessly electrical stimulation effectively promoted the differentiation of NSCs into neurons at the mRNA level. In addition, the expression of GFAP in the graphite+ group increased 1.68‐fold at day 5 and 2.4‐fold at day 10 compared to that in the culture plate group, indicating that graphite patch‐mediated wirelessly electrical stimulation could also slightly promote the differentiation of NSCs into glial cells.

**Figure 4:**
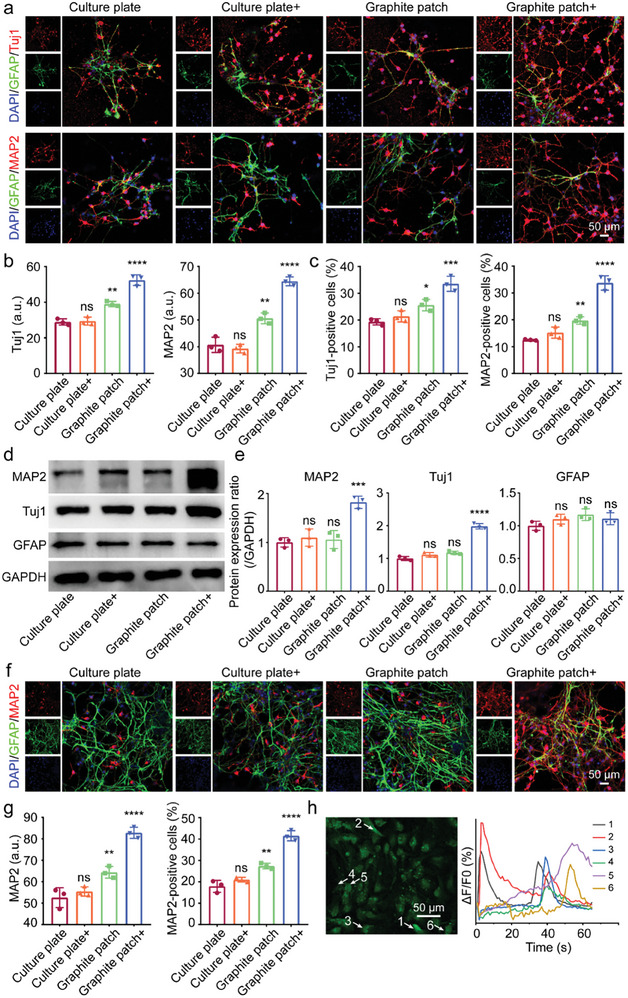
a) Immunofluorescence images of Tuj1, MAP2, and GFAP of NSCs on culture plate or graphite patch without or with (+) a 500‐rpm magnetic field at day 5. Tuj1 and MAP2 was stained red. GFAP was stained green. While nuclei were stained blue by DAPI. b) Fluorescence intensity analysis of Tuj1 and MAP2 based on immunofluorescence staining results in a). c) Evaluation of Tuj1‐positive cells and MAP2‐positive cells based on immunofluorescence staining results in a). d) Western blot results of MAP2, Tuj1, and GFAP of NSCs on culture plate or graphite patch without or with (+) a 500‐rpm magnetic field at day 5. e) The gray value analysis of western blotting results in d). f) Immunofluorescence images of MAP2 and GFAP of NSCs on culture plate or graphite patch without or with (+) a 500‐rpm magnetic field at day 10. g) MAP2 fluorescence intensity analysis and MAP2‐positive cells evaluation at day 10. h) Fluorescence image and the corresponding fluorescence intensity analysis of NSCs cultured on the graphite patch under 500‐rpm magnetic field after stimulation with acetylcholine. The arrows indicate the cells exhibiting the changes in fluorescence intensity. The data were expressed as the mean ± standard deviation (n = 3), as analyzed by a one‐way ANOVA, with Tukey's multiple comparisons test (nsp>0.05, *p<0.05, **p<0.01, ***p<0.001, and ****p<0.0001 compared to the culture plate group).

To further evaluate the influence of graphite patch‐mediated wirelessly stimulation on the neuronal differentiation of NSCs, immunofluorescence staining for Tuj1, MAP2, and GFAP was performed. As shown in **Figure** **4**a, compared to NSCs in the other groups, NSCs on graphite patch with 500‐ rpm magnetic field expressed more Tuj1 and MAP2 and exhibited typical neuronal morphologies and longer axons. We also analyzed the fluorescence intensity of Tuj1 and MAP2 using the Image J software. As shown in Figure 4b, the fluorescence intensity of Tuj1 and MAP2 in the graphite patch+ group was higher than that in the other groups, which was consistent with the q‐PCR results shown in Figure 3. We further calculated the percentage of Tuj1‐ and MAP2‐positive cells (Figure 4c). The ratio of Tuj1‐positive cells in the graphite patch+ group was ≈ 33.5%, while the percentages in the culture plate, culture plate+, and graphite patch groups were only 19.3%, 21.4%, and 25.6%, respectively. The ratio of MAP2‐positive cells in graphite patch+ group was about 33.7%, while the ratios of MAP2‐positive cells in culture plate, culture plate+, and graphite patch group were only 12.5%, 15.2%, and 19.7%, respectively. The above results show that cellularized patch‐mediated wirelessly electrical stimulation significantly promotes neuronal differentiation in the protein level.

Western blot analysis was then performed to confirm the protein expression of Tuj1, MAP2, and GFAP. As shown in Figure 4d, the expression of Tuj1 and MAP2 of NSCs in the graphite patch+ group was the highest among all the groups. We further analyzed the grayscale values of the western blot results using Image J software to semi‐quantitively evaluate protein expression. As shown in Figure 4e, the expression of Tuj1 and MAP2 in the graphite patch+ group increased 2.0‐ and 1.8‐fold, respectively, compared to that in the culture plate group. These results demonstrate that the graphite patch‐mediated wirelessly electrical stimulation driven by the rotating magnetic field promoted the neural differentiation of NSCs. In addition, the electron probe X‐ray microanalyzer (EPMA) shown in Figure S6 (Supporting Information) and cytoskeleton‐staining results in Figure S7 (Supporting Information) also demonstrate that the culture on the graphite patch with 500‐rpm rotating magnetic field exhibited a typical neural morphology.

We further examined the influence of graphite patch‐mediated wirelessly electrical stimulation on the neural differentiation of NSCs by extending the culture time. Figure 4f shows the immunofluorescence staining results for MAP2 (the marker of mature neurons) while Figure 4g shows the corresponding quantitative fluorescence intensity and number of MAP2 positive cells at day 10. The results confirm that more mature neurons with longer axons appeared in the graphite patch+ group than in the other groups. More importantly, the ratio of MAP2‐positive cells in the graphite patch+ group was 41.5%, which was much higher than that in the culture plate (17.9%), culture plate+ (21%), and graphite patch (27.4%) groups. We then examined the neuronal function of differentiated cells using calcium ion fluorescence probe. One typical function of mature neurons is that they can respond to neurotransmitters to transmit signals.^[^ ^27^ ^]^ The presence of certain neurotransmitters generally causes transmembrane ion (e.g., Ca^2+^) flow in neurons.^[^ ^28^ ^]^ Here, we used acetylcholine, a representative neurotransmitter, to evaluate the function of differentiated neurons on graphite patch with a 500‐ rpm rotating magnetic field. As shown in Figure 4h, six of the thirty‐two counted cells (marked with arrows) exhibited obvious fluorescence changes upon stimulation with acetylcholine. In contrast, cells on the graphite patch without rotating magnetic field showed no changes in fluorescence intensity after the addition of acetylcholine (Figure S8, Supporting Information). The above results indicate that the neurons that formed on the graphite patch with wirelessly electrical stimulation had the typical function of mature neurons.

**Figure 5:**
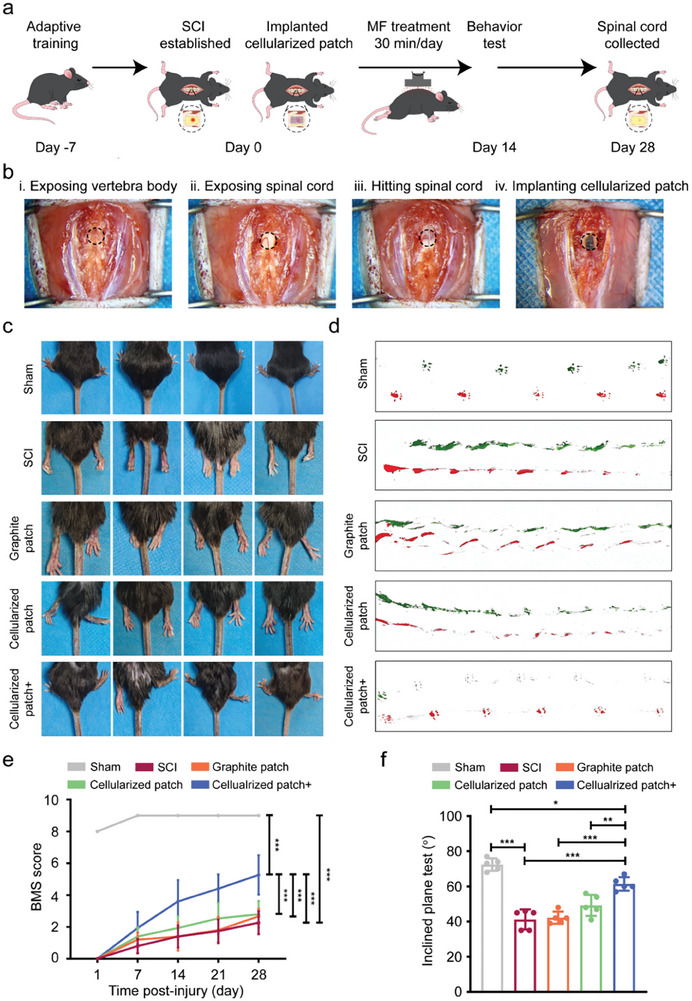
a) Schematic illustration of the use of an electromagnetic cellularized patch for the treatment of SCI mice by leveraging the magnetic field‐driven wirelessly electrical stimulation. b) Digital pictures recorded during construction of the SCI mice model and implantation of the cellularized patch: i) exposing the vertebral body, ii) exposing the spinal cord, iii) hitting the spinal cord, and iv) implanting the cellularized patch to the subarachnoid of spinal cord. c) Digital photos of hind limbs of the mice in sham, SCI, graphite patch, cellularized patch, and cellularized patch+ groups at day 28. d) Digital photos of mouse hind limbs footprints in sham, SCI, graphite patch, cellularized patch, and cellularized patch+ groups at day 28. e) BMS scores for the mice in sham, SCI, graphite patch, cellularized patch, and cellularized patch+ groups at 1‐, 7‐, 14‐, 21‐, and 28‐days post injury. The data were expressed as the mean ± standard deviation (n = 5), as analyzed by a two‐way ANOVA, with Bonferroni's multiple comparisons test (***p<0.001). f) Slope test results for the mice in sham, SCI, graphite patch, cellularized patch, and cellularized patch+ groups at day 28. The data were expressed as the mean ± standard deviation (n = 5), as analyzed by a one‐way ANOVA, with Tukey's multiple comparisons test (*p<0.05, **p<0.01, and ***p<0.001).

The above results demonstrated that the graphite patch‐mediated electrical stimulation promoted neural differentiation and accelerated the maturation of neurons. Directing the differentiation of NSCs into functional neurons has promising applications in the treatment of SCI. We then used the graphite patch seeded with NSCs as an electromagnetic cellularized patch for the treatment of SCI. **Figure** **5**a shows the schematic illustration of the use of cellularized patch to treat SCI mice under a rotating magnetic field. After 7 days of adaptive training, the mice in each group received traumatic SCI, followed by implantation of the cellularized patch into the subarachnoid region of the spinal cord. Figure 5b shows the digital images recorded during the implantation of the cellularized patch. The experiments were divided into 5 groups. For the sham group, only a segment of the vertebra was excised from the mice. In the SCI group, the mice underwent normal SCI surgery. The graphite patch group includes SCI mice implanted with a graphite patch without exogenous NSCs. The cellularized patch group includes SCI mice implanted with a graphite patch seeded with NSCs. In the cellularized patch+ group, SCI mice were implanted with a cellularized patch and treated with a 500‐ rpm magnetic field to apply the wirelessly electrical stimulation. To evaluate the voltage generated on the cellularized patches under a rotating magnetic field in vivo, the tissue (with a thickness of 2–3 mm) from the back to the spinal cord of a mouse was harvested and placed between the magnet bar and the graphite patch (Figure S9a, Supporting Information). The distance between the magnet bar and the tissue was set to 10 mm. Under these conditions, the cellularized patch generated 0.76 mV of electrical signal under a rotating magnetic field of 500 rpm, which reached the voltage range of 0.53–250 mV for promoting the neuronal differentiation of NSCs.^[^ ^29^ ^]^

We examined the hind limbs of the mice 28 days post‐surgery (Figure 5c). In the sham group, the hind limbs of the mice could fully contact the ground, indicating the integrity of the spinal cord. The mice in the SCI group showed significant paralysis as evidenced by the dragging and deviation of the hind limbs, which confirmed the successful establishment of the SCI mouse model. The hind limbs of the SCI mice implanted with graphite patch exhibited conditions similar to those of the SCI mice. In the case of the cellularized patch group, they were able to utilize the instep for ground support, indicating a slight enhancement in the locomotion of their hind limbs. In contrast, for mice in the cellularized patch+ group, their hind limbs showed a certain degree of flexion and ground support, indicating that the SCI was partially recovered when the mice were implanted with the electromagnetic cellularized patch and treated with a rotating magnetic field. Figure 5d shows digital photos of the footprints of the mice 28 days post‐surgery. The footprints of the mice in the sham group were distinct and clear, indicating the synchronized movement of the hind limbs. The SCI mice showed typical disordered footprints due to injury of the spinal cord. The SCI mice implanted with graphite patch or cellularized patch exhibited a slight enhancement in crawling ability. In sharp contrast, the SCI mice implanted with the cellularized patch and treated with a rotating magnetic field (cellularized patch+) exhibited a clearer footprint trace than the other SCI mice, indicating improved crawling ability. The above observations suggest that magnetic cellularized patch under rotating magnetic field could improve the recovery of hind limb motor function.

We then systematically evaluated the recovery of hind limb motor function through Basso Mouse Scale (BMS) scores and slope tests. The BMS is a well‐established open‐field locomotion test with scores ranging from 0 to 9 to assess mouse motor function and SCI recovery.^[^ ^30^ ^]^ Figure 5e shows the weekly BMS scores for mice at 1‐, 7‐, 14‐, 21‐, and 28‐days post‐surgery. In the sham group, the mice stably scored approximately 9 because their motor function was not impaired. For mice in the SCI, graphite patch, and cellularized patch groups, although they displayed notable increases in BMS scores from day 1 to day 28, the scores were still less than 4 at day 28 with no statistically significant variations among these groups (Figure S10, Supporting Information). In contrast, the BMS scores of the mice in the cellularized patch+ group increased from 0 at day 1 to 1.9 at day 7, 3.6 at day 14, 4.4 at day 21, and 5.3 at day 28, which were much higher than the mice in the SCI, graphite patch, and cellularized patch groups, respectively. Furthermore, the BMS of the SCI mice in the cellularized patch+ group remained the highest among all the SCI mice at day 60 (Figure S11, Supporting Information), suggesting that the cellularized patch did not affect the function of the recovered spinal cord. In addition to the BMS, we further evaluated the weight of the hind limbs of the mice via the slope test. Figure 5f shows the slope test results 28 days post‐surgery. The maximal slope angle that the mice in the cellularized patch+ group could bear was 61.4°, which was much higher than that in the SCI (41.2°), graphite patch (42°), and cellularized patch (49.2°) groups.

To directly observe the recovery conditions of the injured spinal cord at the tissue and cellularized level, the spinal cord tissues were harvested 2‐ and 4‐ weeks post‐surgery and sectioned into slices. Notably, the cellularized patch adhered well to the surface of the spinal cord during specimen collection. **Figure** **6**a shows Hematoxylin and Eosin (H&E) staining and Nissl staining of the spinal cord tissues at week 2. The corresponding statistical analysis of the tissue cavities and Nissl densities are shown in Figure 6c. Clearly, compared to those in the Sham group, the mice in the SCI group showed a significant increase in the number of tissue cavities and a decrease in the Nissl density, which was caused by injury to the spinal cord. Implantation of the graphite patch or the cellularized patch led to a slight reduction in the cavity area and a slight increase in Nissl density, indicating that the graphite patch or the cellularized patch could only slightly promote recovery from SCI. In contrast, the SCI mice implanted with the cellularized patch and treated with a rotating magnetic field exhibited a decrease in the area of the vacuole to 22.2% and an increase in the Nissl density to 0.55, while these two values in the SCI group were 58.8% and 0.16, respectively. More importantly, the combination of the cellularized patch and the magnetic field could further reduce the cavity area and improve the Nissl density after 28 days of recovery (Figure S12, S13, Supporting Information). The above results demonstrate that the electromagnetic cellularized patch under a changing magnetic field could improve the recovery of the injured spinal cord.

Figure 6a) H&E staining and Nissl staining of the spinal cord tissues of the mice in sham, SCI, graphite patch, cellularized patch, and cellularized patch+ groups at day 14. b) Immunofluorescence images of the spinal cord tissues of the mice in sham, SCI, graphite patch, cellularized patch, and cellularized patch+ groups at day 14. Tuj1, MBP, and nuclei were stained green, red, and blue, respectively. c) Statistical analysis of the average cavities, Nissl densities, as well as the fluorescence intensities of Tuj1 and MBP based on the H&E, Nissl, and immunostaining results in a,b). The data were expressed as the mean ± standard deviation (n = 5), as analyzed by a one‐way ANOVA, with Tukey's multiple comparisons test (*p<0.05, **p<0.01, and ***p<0.001).

**Figure:**
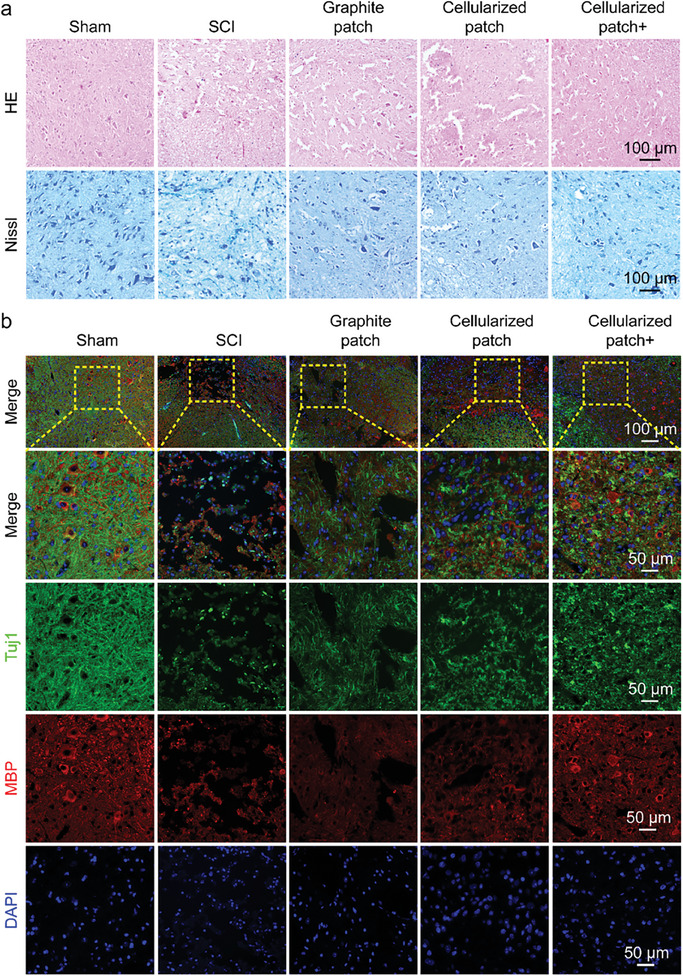

**Figure:**
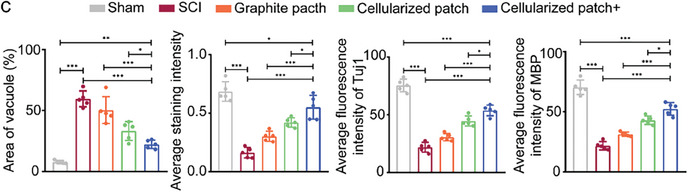

Immunofluorescence staining was performed on the spinal cord tissue slices to further evaluate the recovery of the injured tissues. Figure 6b shows the immunofluorescence images of the spinal cord tissues at the second week, in which Tuj1, myelin basic protein (MBP), and nuclei were stained green, red, and blue, respectively, while the corresponding assessment of the fluorescence intensity is shown in Figure 6c. MBP is a constituent of myelin phosphatide‐affecting factors and contributes to the stability of myelin sheaths within the central nervous system.^[^ ^31^ ^]^ The immunofluorescence staining results show that the expression of Tuj1 and MBP was decreased at the injury site in the spinal cord. The implantation of the graphite patch and cellularized patch slightly restored their expression of these proteins. Moreover, implantation of the cellularized patch together with treatment with a magnetic field significantly improved the expression of both Tuj1 and MBP at the injury site. The fluorescence intensities of Tuj1 and MBP in the SCI group were 21.9 and 21.8, respectively, while those in the cellularized patch+ group were 53.8 and 52.5, respectively. In addition, the expression of Tuj1 and MBP was further enhanced at the fourth week in the cellularized patch+ group (Figures S14, S15, Supporting Information).

Notably, the expression of GFAP was also slightly enhanced in the graphite patch, cellularized patch, and cellularized patch+ groups (Figures S16, S17, Supporting Information). GFAP is related to astrocytes. Up to today, whether astrocytes are beneficial for regeneration after SCI is doubtable.^[^ ^32^ ^]^ In any case, in our study, the slightly increased expression of GFAP in the cellularized patch group did not influence the repair of the SCI. The above results confirm that the electromagnetic cellularized patch capable of generating wirelessly electrical stimulation under magnetic promoted myelin formation, neural regeneration, and tissue repair in spinal cord injuries. It is worth to mention that the implantation of the graphite patch without NSCs did not improve the recovery of the injured spinal cord under the magnetic treatment, indicating that the enhanced neuronal differentiation of exogenous NSCs on the electromagnetic cellularized patch promoted the repair of SCI.

To confirm whether the transplanted NSCs were differentiated into neurons in vivo, Green fluorescent protein (GFP)‐labeled NSCs (obtained from GFP transgenic mice) instead of normal NSCs were seeded on the graphite patch and then used for the injured spinal cord repair. GFP‐labeled NSCs appeared at the lesion site in both the cellularized patch group and the cellularized patch+ group at day 14 and 28 (Figures S18 and S20, Supporting Information), indicating that the implanted NSCs survived well in vivo.

More importantly, the GFP intensity and degree of colocalization of GFP and Tuj1 were greater in the cellularized patch+ group than in the cellularized patch group (Figure S19 and S21, Supporting Information), indicating that the cellularized patch could promote the differentiation of NSCs in vivo under magnetic field treatment. The above results demonstrate that the wireless electrical stimulation generated by the cellularized patch promoted the neuronal differentiation of transplanted cells in vivo, which further promoted the repair of SCI. It is worth to mention that the cellularized patch was not removed from the mouse after treatment and was kept at the subarachnoid site to serve as a protective layer for immobilizing and protecting the spinal cord. Keeping the patch at the subarachnoid site did not cause obvious inflammation in the spinal cord, brain, heart, liver, spleen, lung, or kidney (Figure S22, Supporting Information), indicating that the patch had good biocompatibility in vivo. More importantly, our research aims to repair injured spinal cord tissue by promoting the neuronal differentiation of implanted NSCs by leveraging the cellularized patch‐mediated wireless electrical stimulation, which is different from previous reports related to direct electrical stimulation of the spinal cord. For example, Courtine et al. reported a wireless “brain‐spinal cord interface” to bypass the injured lesion of the spinal cord and transmit the signals from the motor cortex to the lumbar spinal cord. Although this approach restored the conscious movements of a temporarily paralyzed leg caused by SCI of rhesus monkeys, the injured spinal cord was not repaired or regenerated.^[^ ^33^ ^]^ In addition, direct electrical stimulation for SCI treatment generally requires an electrical signal of 0.8–9 V,^[^ ^34^ ^]^ while inducing the neuronal differentiation of NSCs requires only a much lower (0.53–250 mV) electrical signal.^[^ ^29^ ^]^

## 3 Conclusion

In summary, we prepared a patch composed of slightly flaky graphite nanosheets and NSCs. The graphite patch could generate wirelessly electrical signals under a rotating magnetic field. The intensity and frequency of the generated electrical signals could be controlled by adjusting the rotation speed of the magnetic field. Cellularized patch‐mediated wirelessly electrical stimulation promoted the rate and proportion of the NSCs that differentiated into functional mature neurons. After implantation into the subarachnoid site of the injured spinal cord of the SCI mice, the cellularized patch improved the recovery of the injured spinal cord under the rotating magnetic field. This electromagnetic cellularized patch holds great promise for the remote treatment of SCI and other diseases of the central and peripheral nervous systems.

## 4 Experimental Section

### Materials

Graphite nanosheets was purchased from XFNano (China, XF011, 7782‐42‐5). Neurobasal medium (21 103 049), glutaMAXTM‐1 (35 050 061), fetal bovine serum (FBS, A5669701), Bmur27 supplement (B27, 17 504 044), and penicillin‐streptomycin (15 070 063) were purchased from Gibco (USA). Epidermal growth factor (EGF, 315‐09) and basic fibroblast growth factor (bFGF, 450‐33) were purchased from PeproTech (USA). PBS (P1020), paraformaldehyde (P1110), Triton X‐100 (T8200), Rhodamine Phalloidin (CA1610), and RIPA lysis buffer (R0020) were purchased from Solaribio (China). Propidium iodide (PI, 25535‐16‐4), calcein AM (148504‐34‐1), and bovine serum albumin (BSA, 9048‐46‐8) were purchased from Sigma‐Aldrich (USA). CCK‐8 (CK04) was purchased from Dogindo (Japan). Trizol reagent (R401‐01) was purchased from Vazyme (China). Fluo‐4 AM (S1060) and BCA Protein Assay Kit (P0012) were purchased from Beyotime (China). The RT‐qPCR primers of Nestin, Tuj1, GFAP, and MAP2 were designed and ordered from Biosune (China). DAPI (ab285390), the first, and second antibodies against Tuj1, GFAP, MAP2, and MBP (see Table S1, Supporting Information for the identifiers and the type of antibody) were purchased from Abcam (UK).

### Graphite Patch Fabrication and Characterization

The 0.6 mg of graphite nanosheets was dispersed in 10 mL of ultra‐pure water under sonication to obtain a 60 µg mL ^−1^graphite dispersion. Then, the graphite patch with a diameter of 3 cm and thickness of 200 µm was obtained by draining 50 mL of graphite dispersion on a filter and vacuum drying. The morphology and structure were characterized by using SEM (S‐4800, Hitachi, Japan), AFM (Icon, Bruker, USA), and Raman spectrometer (LabRAM HR Evolution, France).

### Wirelessly Electrical Stimulation Setup

The rotating magnetic field was provided by the Neodymium‐iron‐boron permanent magnet connected to the agitator motor. Two magnets were installed on the agitator and rotated with the motor shaft to provide a rotating magnetic field. The changing frequency of the magnetic field can be regulated by adjusting the rotation speed of the motor. A plastic bracket with a distance of 1 cm from the magnet was installed above the magnet to support the culture plate and graphite patch. A CH‐1800 gaussmeter (Reanow, China) was used to measure the magnetic field intensity at 1 cm above the magnet. The output voltage of the culture plate and the graphite patch was measured by the oscilloscope (Keysight infiniivision dsox3034t, USA) at 1 cm above the magnet under different magnetic field rotation speeds (0, 100, 200, 300, 400, 500 rpm). The electrical signals generated on the graphite patch under a tissue was measured by the oscilloscope under a rotating magnetic field of 500 rpm. The tissue (with a thickness of 2–3 mm) from the back to the spinal cord of a mouse was harvested and placed between the magnet bar and the graphite patch to mimic the in vivo state of the cellularized patch. The distance between the magnet bar and the tissue was set to be 10 mm.

### Isolation, Culture, and Differentiation of NSCs

The NSCs were isolated from the C57/BL6 mouse embryos at embryonic days 12–14 (Beijing Vital River Laboratory Animal Technology, China) by following the previous protocol.^[^ ^35^ ^]^ The GFP transgenic mouse were obtained from Shanghai Model Organisms Center, Inc. The NSCs were cultured in neurobasal medium supplemented with 2% B27 supplement, 1% GlutaMAX^TM^‐1, 20 ng mL^−1^ of mEGF, 20 ng mL^−1^ of mbFGF, and 1% penicillin/streptomycin, and maintained in humidified air with 5% CO_2_ at 37 °C. For the differentiation of NSC, the culture medium was switched to the neurobasal medium supplemented with 2% B27 supplement, 1% glutaMAX^TM^‐1, 1% FBS, and 1% penicillin/streptomycin.

### SEM and EPMA Imaging

NSCs of 5 × 10^5^ were cultured on graphite patches with a diameter of 1.3 cm or culture plates under the rotating magnetic field for 3 or 5 days. After washing with PBS 3 times, the cells were fixed with 2.5% glutaraldehyde room temperature for 30 min, followed by dehydration with different concentrations (30%, 50%, 70%, 80%, 90%, 95%, 100%) of ethanol. The samples were then freeze‐dried using a lyophilizer before being observed under SEM (ZEISS Gemini SEM 500) and EPMA‐1720H.

### Live/Dead Staining and CCK‐8 Test

For Live/Dead staining, 5 × 10^5^ of NSCs were seeded on the graphite patches with a diameter of 1.3 cm or in 24‐well culture plates with or without rotating magnetic field for 2 days. Afterward, changing magnetic field provided by the permanent magnet (80 mT) on a rotator (500 rpm) was applied to the cells at a distance of 1 cm for 15 min every day. After culturing for 72 h, 300 µL of neurobasal medium containing 0.5 µM of calcein‐AM and 3 µM of PI was used to replace the culture medium, followed by incubation for 30 min at 37 °C. The samples were observed using the CLSM (Zeiss Co, Germany). The live and dead cells were counted using the software Image J to calculate the survival rate. For CCK‐8 test, 5 × 10^5^ of NSCs were seeded on the graphite patches (Diameter:1.3 cm) or in 96‐well culture plates with or without rotating magnetic field, followed by incubation for 1, 2, or 3 days, respectively. The changing magnetic field was applied to the cells for 15 min every day. Then 10 µL of CCK‐8 solution was added into the culture medium each well, followed by incubation for 1 h at 37 °C. The level of water‐soluble formazan was assayed at a wavelength of 450 nm using a microplate reader (Multiscan MK3, Thermo, USA). Three parallel replicates were used for each data point.

### Immunofluorescence Staining

NSCs of 5 × 10^5^ were seeded on the graphite patches with a diameter of 1.3 cm or in 24‐well culture plates with or without rotating magnetic field for 5 and 10 days. After washing with PBS three times, the NSCs were fixed with 4% paraformaldehyde at room temperature for 20 min. Then the samples were permeabilized by 0.1% Triton X‐100 for 10 min and blocked with 1% BSA for 30 min at room temperature. Afterward, the cells were incubated with primary antibodies (diluted by 800 times for Tuj1, MAP2, and 1000 times for GFAP) in 1% BSA at 4 °C overnight, and the secondary antibody (diluted by1000 times) at room temperature for 1 h. After washing with PBS three times, the cells were incubated with 1 µg mL^−1^ of DAPI for 5 min to stain the nuclei. The samples were imaged with CLSM. The expressions of Tuj1 and MAP2 proteins was semi‐quantified based on the fluorescence intensities using the software Image J.

### Cytoskeleton Staining

NSCs of 5 × 10^5^ were seeded on the graphite patches with a diameter of 1.3 cm or in 24‐well culture plates with or without rotating magnetic field for 5 days. After washing with PBS 3 times, the NSCs were fixed with 4% paraformaldehyde for 20 min at room temperature. Then the samples were permeabilized by 0.1% Triton X‐100 for 10 min at room temperature. Afterward, the cells were incubated with Rhodamine Phalloidin (diluted by1000 times) for 1 h at room temperature. After washing with PBS 3 times, the cells were incubated with 1 µg mL^−1^ of DAPI for 5 min to stain the nuclei. The samples were imaged with CLSM.

### Real‐Time Quantitative Polymerase Chain Reaction (RT‐qPCR)

NSCs of 1 × 10^6^ were seeded on the graphite patches with a diameter of 1.8 cm or in 12‐well culture plates with or without rotating magnetic field for 5 and 10 days. The NSCs were treated with Trizol Reagent to extract the total RNA. The concentration, purity, and integrity of the extracted RNA were determined using a Q‐5000 spectrophotometer (Quawell, Q‐5000, America) at 260/280 nm. The qPCR analysis was performed using the LightCyler@96 SW 1.1 software to analyze the expressions of the housekeeping gene, GAPDH, and the four genes of Nestin, Tuj1, GFAP, and MAP2 (see Table S2, Supporting Information for primer sequence). The relative expression level of the target gene was normalized to GAPDH. Three parallel replicates were used for each data point.

### Western Blot

NSCs of 2 × 10^6^ were seeded on the graphite patches with a diameter of 3 cm or in 6‐well culture plates with or without rotating magnetic field for 5 days. The total protein of cells was extracted by protein extraction reagent (RIPA lysis buffer). The concentration of the extracted protein was determined using BCA Protein Assay Kit. The protein samples were then analyzed using SDS‐polyacrylamide gel electrophoresis (PAGE). Afterward, the proteins were transferred from the PAGE gel onto polyvinylidene difluoride (PVDF) membranes. After blocking with 5% skim milk powder at room temperature for 1 h, the membranes were incubated with primary antibodies (diluted by 1000 times for MAP2, 5000 times for Tuj1, and 2000 times for GFAP, respectively) at 4 °C overnight and secondary antibodies at room temperature for 1 h. The samples were then treated with ECL plus reagents and imaged under the chemiluminescence system (Vazyme, China). The expression of the targeted proteins was semi‐quantified with software Image J.

### Calcium Imaging of NSCs

NSCs of 5 × 10^5^ were seeded on the graphite patches with a diameter of 1.3 cm with or without rotating magnetic field for 5 days. The NSCs were washed with PBS 3 times, followed by incubation with 500 µL of Fluo‐4 AM working solution (2 µg mL^−1^) at 37 °C for 10 min. The neurotransmitter (90.8 mg mL^−1^ of Ach) was used to stimulate the cells, followed by the observation of the fluorescence changes using CLSM. The fluorescence changes of each cell were analyzed using software Image J.

### Animal Experiment

All animal care and experiments followed the National Institutes of Health Guide for the care and use of laboratory animals and were approved by the Animal Care Committee of the First Affiliated Hospital of Shandong First Medical University (Approval No. SYDWLS2021020). In accordance with the prior report, a mouse SCI model was established.^[^ ^36^ ^]^ Briefly, male C57BL/6J mice (8‐10 weeks old, n = 125) were provided by Jinan Pengyue Experimental Animal Breeding Co., Ltd. (Jinan, Shandong Province; Nos. SCXK (LU) 2022‐0006). The mice were randomly divided into 5 groups. Sham, SCI, Graphite patch, Cellularized patch, and Cellularized patch+ groups. In the experiment aimed at identifying the source of differentiated cells consisted of four groups: Sham, SCI, Cellularized patch, and Cellularized patch+ groups. And the biosafety experiment comprised 2 groups, namely Sham and Graphite patch group (intact spinal cord). Five parallel replicates were used for each data point.

Pentobarbital sodium (50 mg k^−1^g) was injected into the abdominal cavity to anesthetize the mice. After clear exposure of the T9‐T11 vertebras by tissue scissors, and T10 vertebra was removed. Then the T10 spinal cord was completely exposed, the spinal cord was hit with 70 kilodyne by KW‐ZJ automatic craniocerebral spinal impingement instrument (NJKEWBIO, Nanjing, China).^[^ ^37^ ^]^ For the graphite patch group, a thin graphite film (1 mm × 5 mm) was implanted to the subarachnoid of the spinal cord and make it to the surface of the injured spinal cord. For the cellularized patch and cellularized patch+ groups, a thin graphite film (1 mm × 5 mm) loaded with NSCs (5 × 10^6^) was implanted into the spinal subarachnoid site and kept on the surface of the injured spinal cord. The patch was sutured to the spinal dura mater using a 10‐0 surgical absorbable suture with the assistance of a stereo microscope (Figure S23, Supporting Infromation). Subsequently, the muscle, subcutaneous tissue, and skin were sutured in layers. For the cellularized patch+ group, the changing magnetic field provided by the permanent magnet (80 mT) on a rotator (500 rpm) was applied to the mice at a distance of 1 cm for 30 min every day for two weeks. As shown in Figure S24 (Supporting Information), to avoid the movement of the mouse, the mouse was putted in an animal fixation apparatus when treating the mouse with the magnetic field. In addition, the distance between the magnet and the mouse was kept at 1 cm to avoid the fluctuation of the magnetic treatment. Urination and feeding were performed daily for all groups. For evaluation of the in vivo biocompatibility, the graphite patch were implanted to the subarachnoid after full exposure of the T10 spinal cord without injury treatment.

### Assessment of Motor Function

At one, two, three, four‐weeks, and 60‐days post‐surgery, the BMS locomotor rating scale was used to assess the motor abilities of the mice.^[^ ^38^ ^]^ Slope testing was also conducted to evaluate the motor capabilities at week four.^[^ ^39^ ^]^ By altering the angle of the slope, the ability of the mice to remain on the slope for 5 seconds without falling was observed.

### Histological Evaluation, Nissl Staining, and Immunofluorescence Staining

At two‐ and four‐weeks post‐surgery, mice were deeply anesthetized, euthanized, and subsequently perfused with physiological saline followed by 4% paraformaldehyde at room temperature for 30 min. The spinal cord tissue at the lesion site was collected ≈approximately 2 cm from the lesion site. And the vital organs (including heart, liver, spleen, lung, kidney, brain and spinal cord) were harvested at day 28 for biosafety assessment. The collected tissue was embedded in paraffin and cut into 4 µm sections using a paraffin microtome.

For histological evaluation, the sections were deparaffinized and washed in water, followed by staining with 0.5% hematoxylin for 3 minutes. After treating with hematoxylin differentiation solution for 5 seconds and washing with water, the sections were then stained with 0.5% eosin for 3 minutes, followed by dehydration before mounting. Subsequently, the sections were observed using an inverted microscope and the cavity area was estimated using Image Pro Plus software.

For Nissl staining, the sections were deparaffinized, washed in water, and then stained with 0.5% Nissl staining solution for 3 minutes. The sections were then dehydrated, cleared with xylene, and mounted, followed by observation using an inverted microscope.^[^ ^40^ ^]^ Subsequently, the Image Pro Plus software was employed for the analysis of Nissl staining.

For immunofluorescence staining of tissue sections, after dewaxing and antigen repair, the sections were incubated overnight at 4 °C with primary antibodies (diluted by 100 times for Tuj1, MBP, MBP, and GFAP, Cell Signaling Technology, USA). After washing 3 times with PBS, the tissue sections were incubated with the secondary antibodies at room temperature for 1 h before staining with DAPI. The samples were imaged with CLSM. The expressions of Tuj1, MAP2, and GFP proteins were semi‐quantified based on the fluorescence intensities using the software Image J.

At the conclusion of this study, the surviving mice were humanely euthanized, and tissues spanning from the dorsal region to the spinal cord (excluding the spinal column) were collected for subsequent electrical signal analysis.

### Statistical Analysis

The data were presented as the mean ± standard deviation. Statistical analysis was performed using GraphPad Prism software by one‐way analysis of variance (ANOVA) and Tukey's multiple comparisons test or two‐way ANOVA and Bonferroni's multiple comparisons test. Statistical significance was accepted at *p<0.05, **p<0.01, ***p<0.001, and ****p<0.0001. The above information as well as the number of replicates and animals were indicated in each figure legends.

## Conflict of Interest

The authors declare no conflict of interest.

## Supporting information

Supporting Information

## Data Availability

The data that support the findings of this study are available from the corresponding author upon reasonable request.
