# Supplementary material for: Electromagnetic Cellularized Patch with Wirelessly Electrical Stimulation for Promoting Neuronal Differentiation and Spinal Cord Injury Repair
Source: Adv Sci (Weinh). 2024 Jun 13;11(30):2307527. doi: 10.1002/advs.202307527 (PMC11321663; doi:10.1002/advs.202307527)
Supplement: Supplementary file 1 — Supporting Information [file ADVS-11-2307527-s001.pdf]

ADVANCED SCIENCE
Open Access

Supporting Information

for *Adv. Sci.*, DOI 10.1002/advs.202307527

Electromagnetic Cellularized Patch with Wirelessly Electrical Stimulation for Promoting Neuronal Differentiation and Spinal Cord Injury Repair

Liang Wang, Hongbo Zhao, Min Han, Hongru Yang, Ming Lei, Wenhan Wang, Keyi Li, Yiwei Li, Yuanhua Sang, Tao Xin*, Hong Liu* and Jichuan Qiu**

Supporting Information

Electromagnetic Cellularized Patch with Wirelessly Electrical Stimulation for Promoting Neuronal Differentiation and Spinal Cord Injury Repair

Liang Wang†, Hongbo Zhao†, Min Han, Hongru Yang, Ming Lei, Wenhan Wang, Keyi Li, Yiwei Li, Yuanhua Sang, Tao Xin*, Hong Liu*, and Jichuan Qiu**

L. Wang, H. Yang, M. Lei, W. Wang, K. Li, Y. Li, Prof. Y. Sang, Prof. H. Liu, Prof. J. Qiu

State Key Laboratory of Crystal Materials, Shandong University, Jinan, Shandong, 250100, P. R. China

E-mail: jichuan.qiu@sdu.edu.cn, hongliu@sdu.edu.cn, sangyh@sdu.edu.cn

Prof. H. Liu

Institute for Advanced Interdisciplinary Research, University of Jinan, Jinan, Shandong, 250022, P. R. China

E-mail: hongliu@sdu.edu.cn

H. Zhao, Prof. M. Han, Prof. T. Xin.

Department of Neurosurgery, The First Affiliated Hospital of Shandong First Medical University & Shandong Provincial Qianfoshan Hospital, Jinan 250014, P. R. China

E-mail: drxintao@yeah.net

Prof. T. Xin.

Department of Neurosurgery, Shandong Provincial Qianfoshan Hospital, Shandong University, Jinan 250014, P. R. China

Medical Science and Technology Innovation Center, Shandong First Medical University and Shandong Academy of Medical Sciences, Jinan 250117, P. R. China

Department of Neurosurgery, Jiangxi Provincial People's Hospital, Nanchang, Jiangxi 330006, P. R. China

E-mail: drxintao@yeah.net

†These authors contribute equally to the preparation of this article.

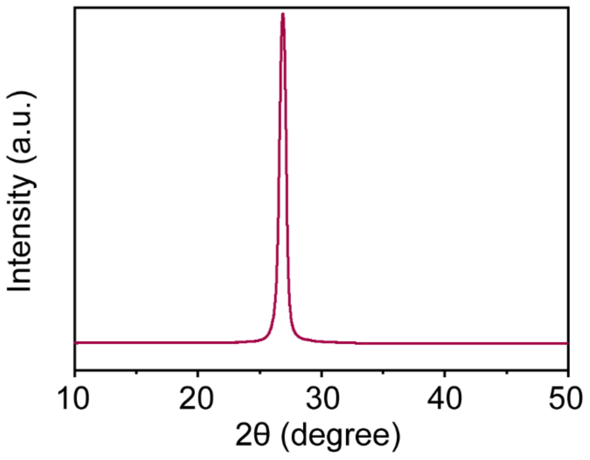

Figure S1. XRD pattern of the graphite patch.

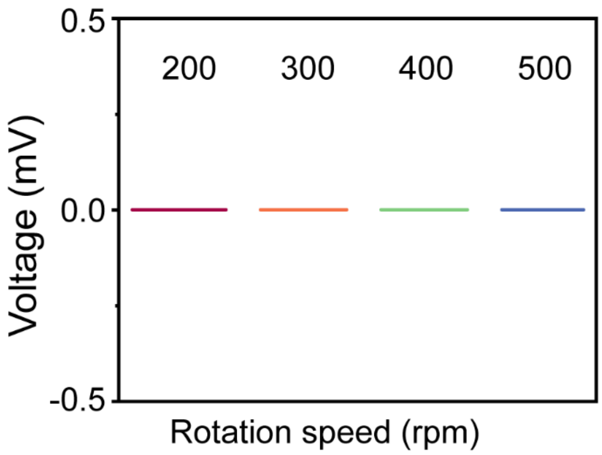

Figure S2. The voltage generated on the culture plate when placed under a rotating magnetic field at rotation speeds of 200, 300, 400, and 500 rpm.

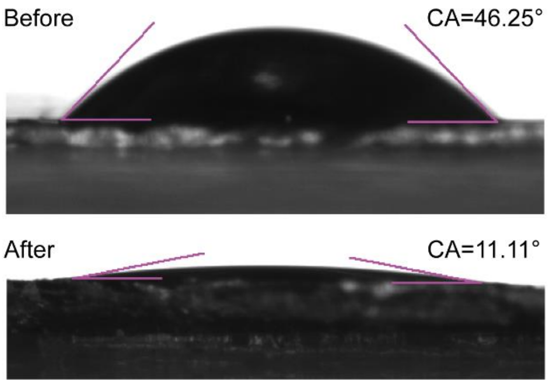

Figure S3. Water contact angles of graphite patch before and after oxygen plasma treatment.

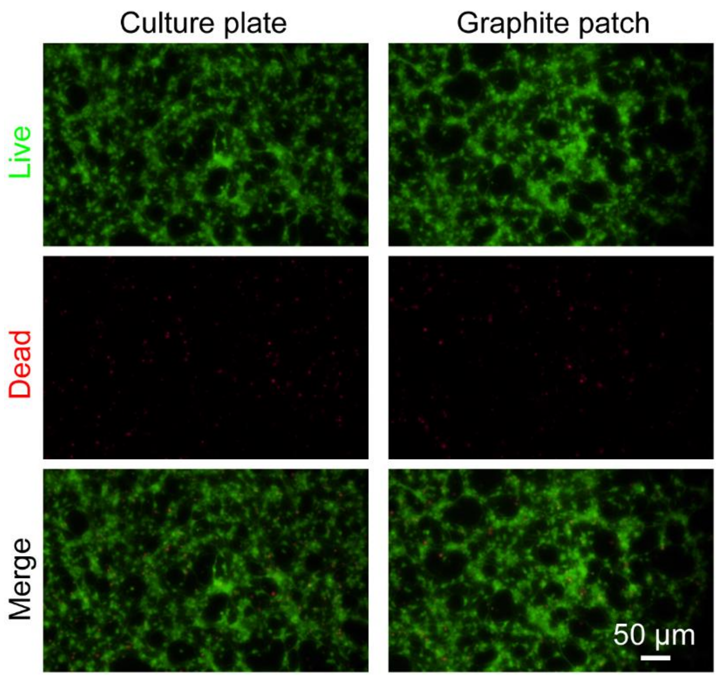

Figure S4. Live/Dead staining of NSCs cultured on the culture plate or graphite patch for 2 days. Living and dead cells were stained green and red, respectively.

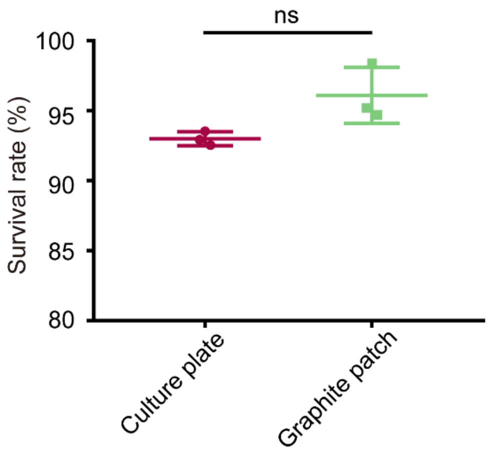

Figure S5. Survival rate of NSCs culture on the culture plate or graphite patch for 2 days based on the Live/Dead staining in Figure S4. The data were expressed as the mean ± standard deviation (n=3), as analyzed by a one-way ANOVA, with Tukey's multiple comparisons test (nsp>0.05).

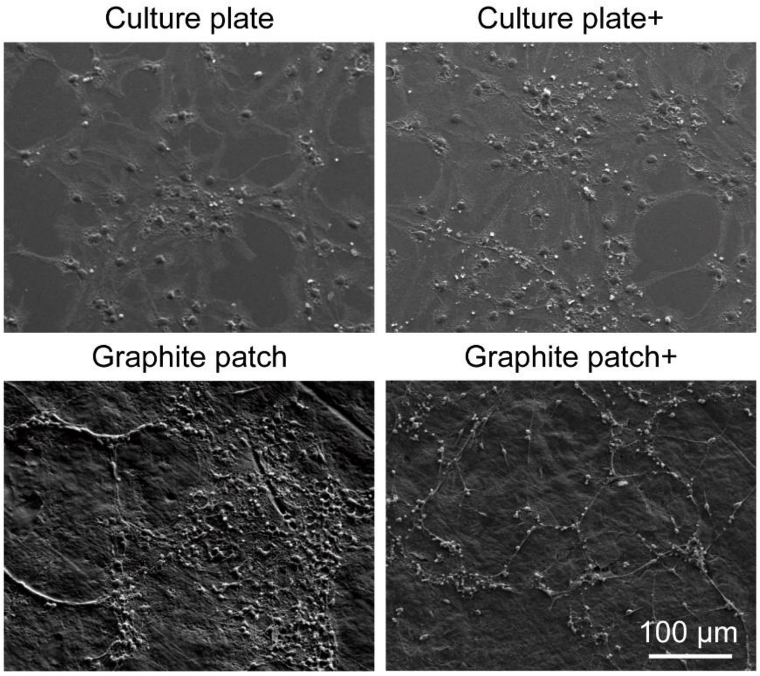

Figure S6. EPMA images of NSCs on culture plate or graphite patch without or with (+) a 500-rpm magnetic field at day 5.

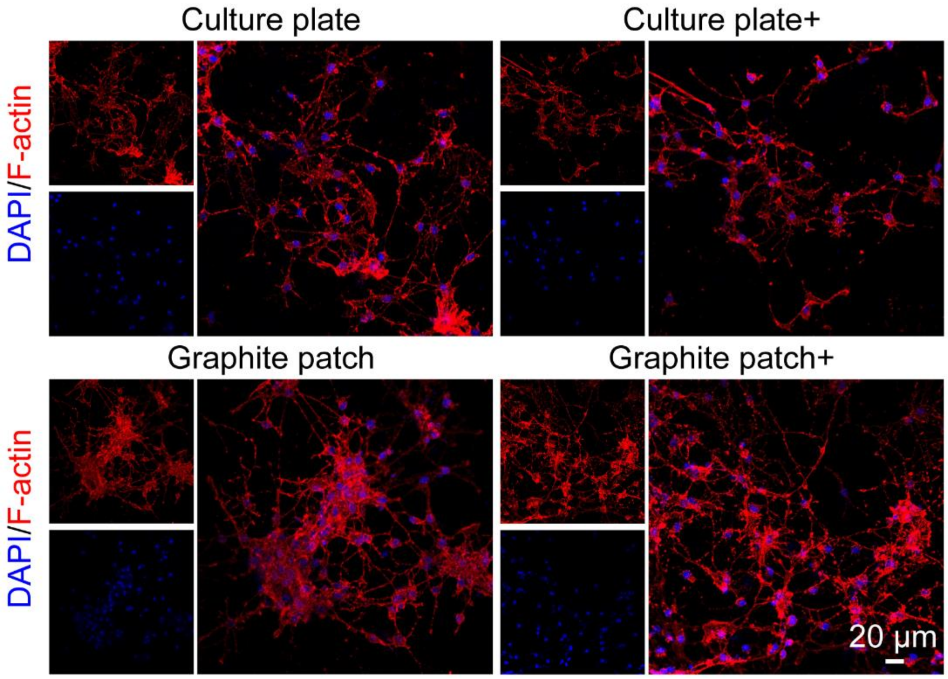

Figure S7. Cytoskeleton staining of NSCs on culture plate or graphite patch without or with (+) a 500-rpm magnetic field at day 5. F-Actin was stained red by phalloidin while nuclei were stained blue by DAPI.

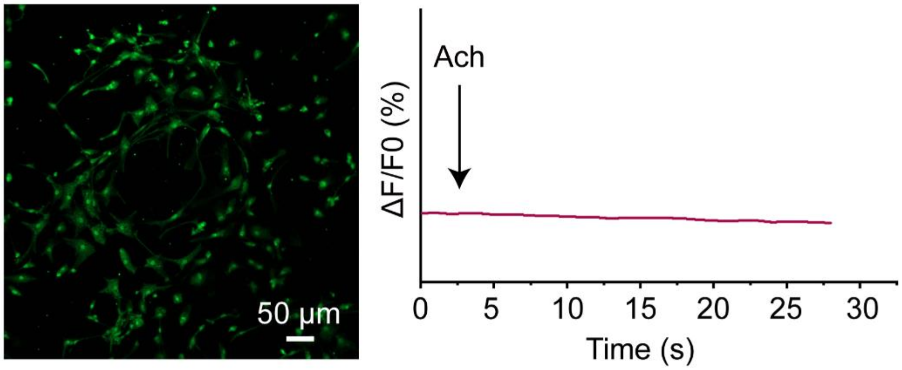

Figure S8. Fluorescence image and the corresponding fluorescence intensity analysis of NSCs cultured on the graphite patch without rotating magnetic field for 5 days after stimulation with acetylcholine.

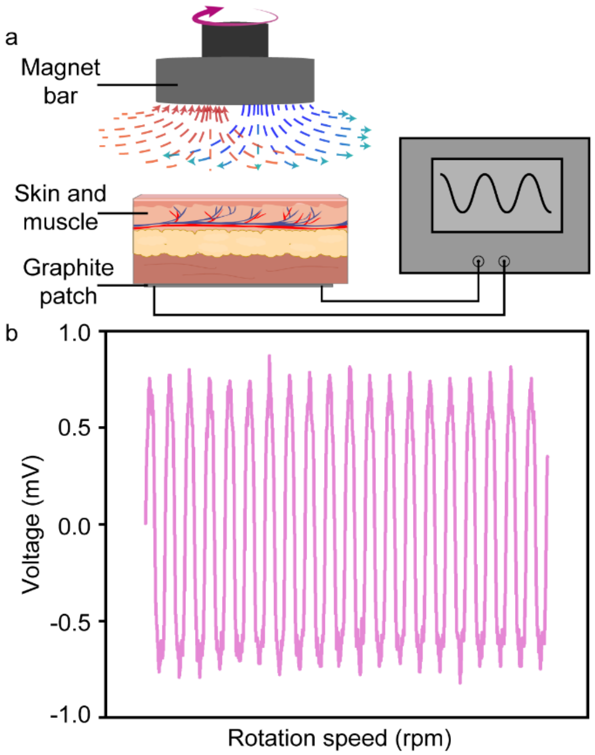

Figure S9. a) Schematic showing the detection of the electrical signal generated on the graphite patch by placing a tissue between the magnet bar and the graphite patch to mimic the in vivo state of the cellularized patch. The tissue with a thickness of 2-3 mm was harvested from the back to the spinal cord of a mouse. The distance between the magnet bar and the tissue was set to be 10 mm. b) The voltage generated on the graphite patch under a rotating magnetic field at a rotation speed of 500 rpm.

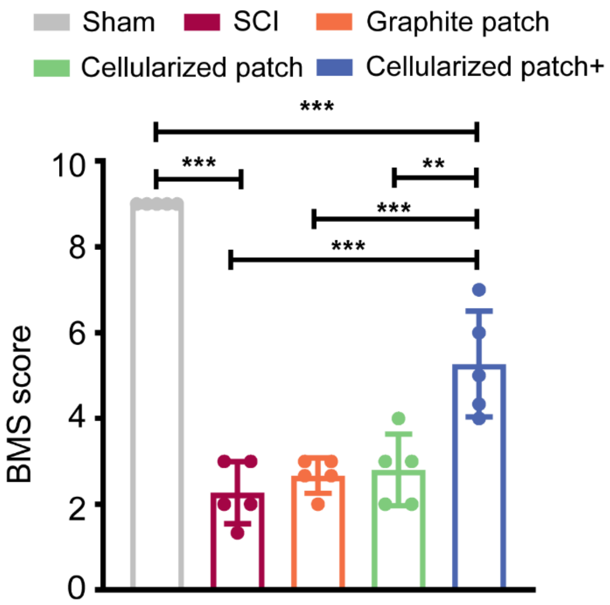

Figure S10. BMS scores for the mice in sham, SCI, graphite patch, cellularized patch, and cellularized patch+ groups at 28-days post injury. The data were expressed as the mean ± standard deviation (n=5), as analyzed by a one-way ANOVA, with Tukey's multiple comparisons test (**p<0.01 and ***p<0.001).

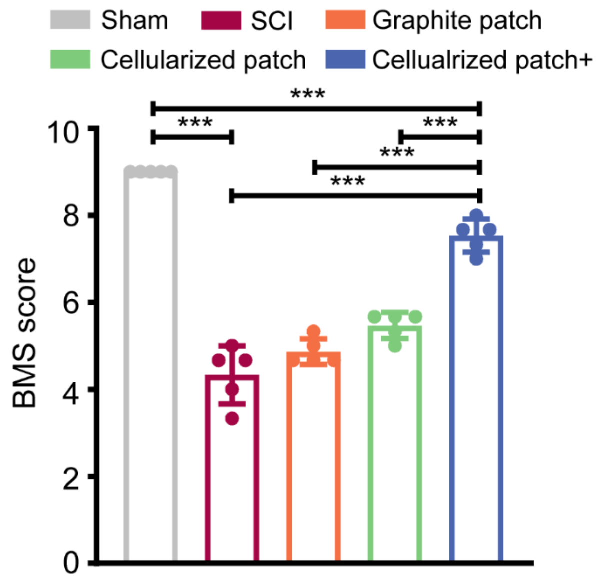

Figure S11. BMS scores for the mice in sham, SCI, graphite patch, cellularized patch, and cellularized patch+ groups at 60-days post injury. The data were expressed as the mean ± standard deviation (n=5), as analyzed by a one-way ANOVA, with Tukey's multiple comparisons test (***p<0.001).

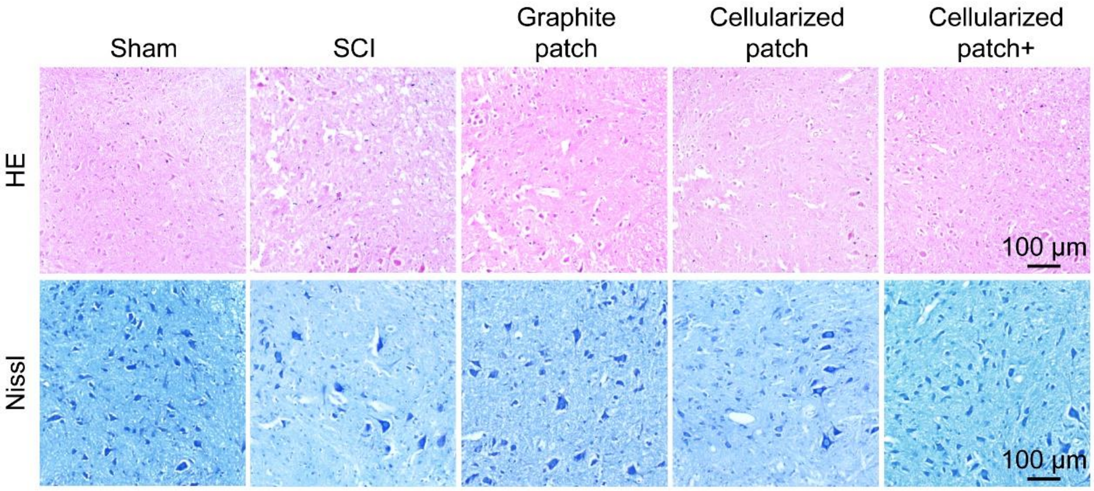

Figure S12. H&E staining and Nissl staining of the spinal cord tissues of the mice in sham, SCI, graphite patch, cellularized patch, and cellularized patch+ groups at day 28.

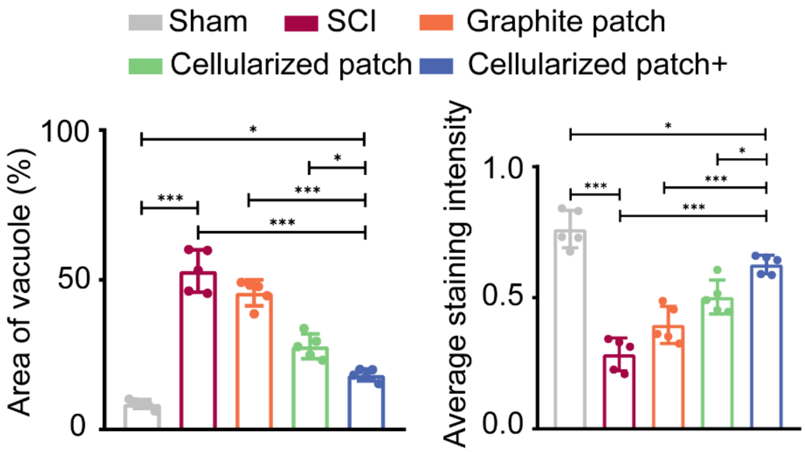

Figure S13. Statistical analysis of the average cavities and Nissl densities at day 28 based on the HE& Nissl results in Figure S12. The data were expressed as the mean ± standard deviation (n=5),

as analyzed by a one-way ANOVA, with Tukey's multiple comparisons test (*p<0.05 and ***p<0.001).

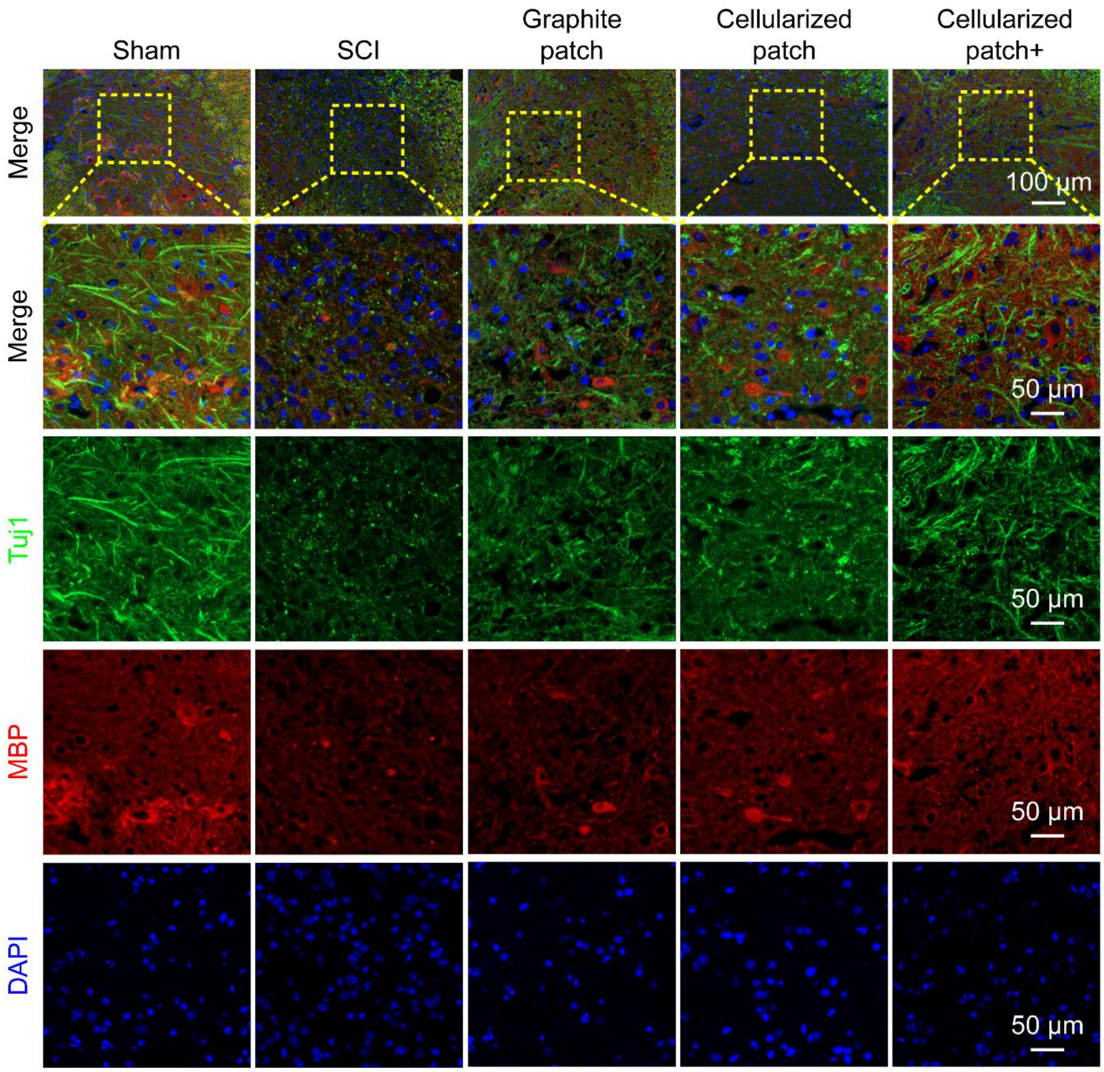

Figure S14. Immunofluorescence images of the spinal cord tissues of the mice in sham, SCI, graphite patch, cellularized patch, and cellularized patch+ groups at day 28. Tuj1, MBP, and nuclei were stained green, red, and blue, respectively.

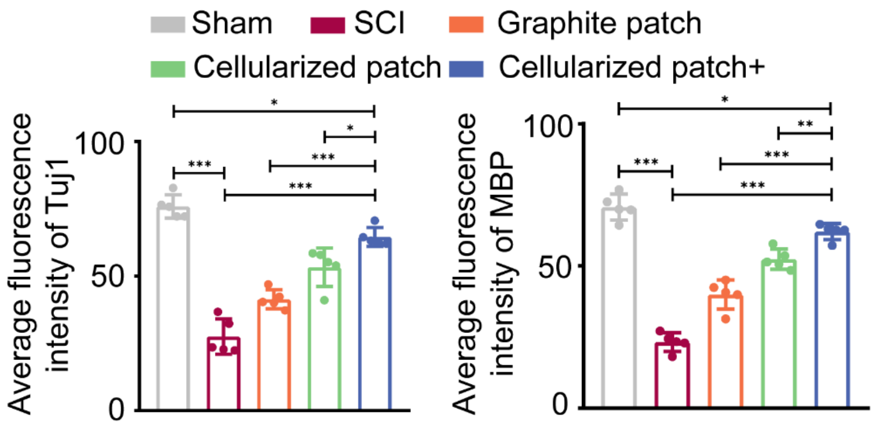

Figure S15. Statistical analysis of the fluorescence intensities of Tuj1 and MBP at day 28 based on the immunostaining results in Figure S14. The data were expressed as the mean ± standard deviation (n=5), as analyzed by a one-way ANOVA, with Tukey's multiple comparisons test (*p<0.05, **p<0.01, and ***p<0.001).

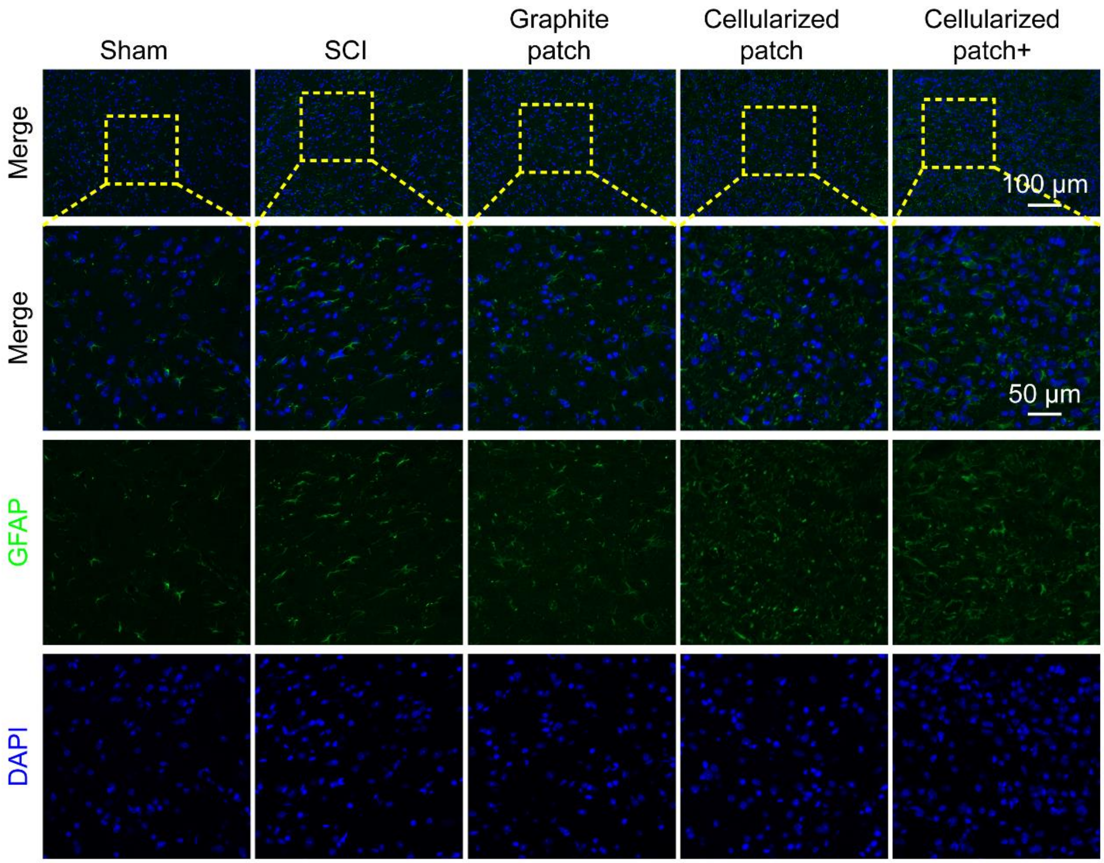

Figure S16. Immunofluorescence images of the spinal cord tissues of the mice in sham, SCI, graphite patch, cellularized patch, and cellularized patch+ groups at day 14. GFAP and nuclei were stained green and blue, respectively.

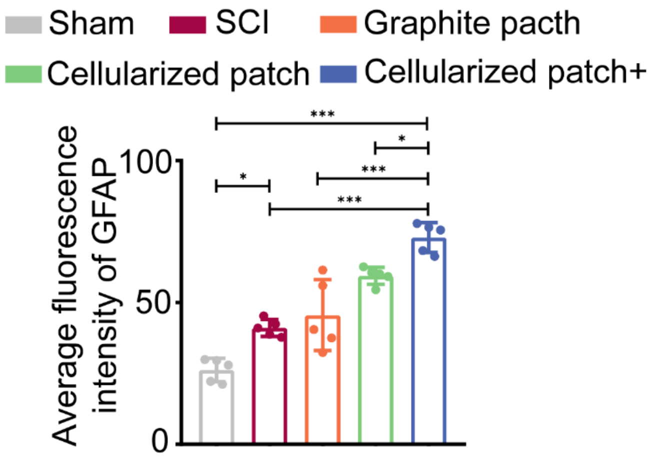

Figure S17. Statistical analysis of the fluorescence intensities of GFAP at day 14 based on the immunostaining results in Figure S16. The data were expressed as the mean ± standard deviation (n=5), as analyzed by a one-way ANOVA, with Tukey's multiple comparisons test (*$p<0.05$ and ***$p<0.001$).

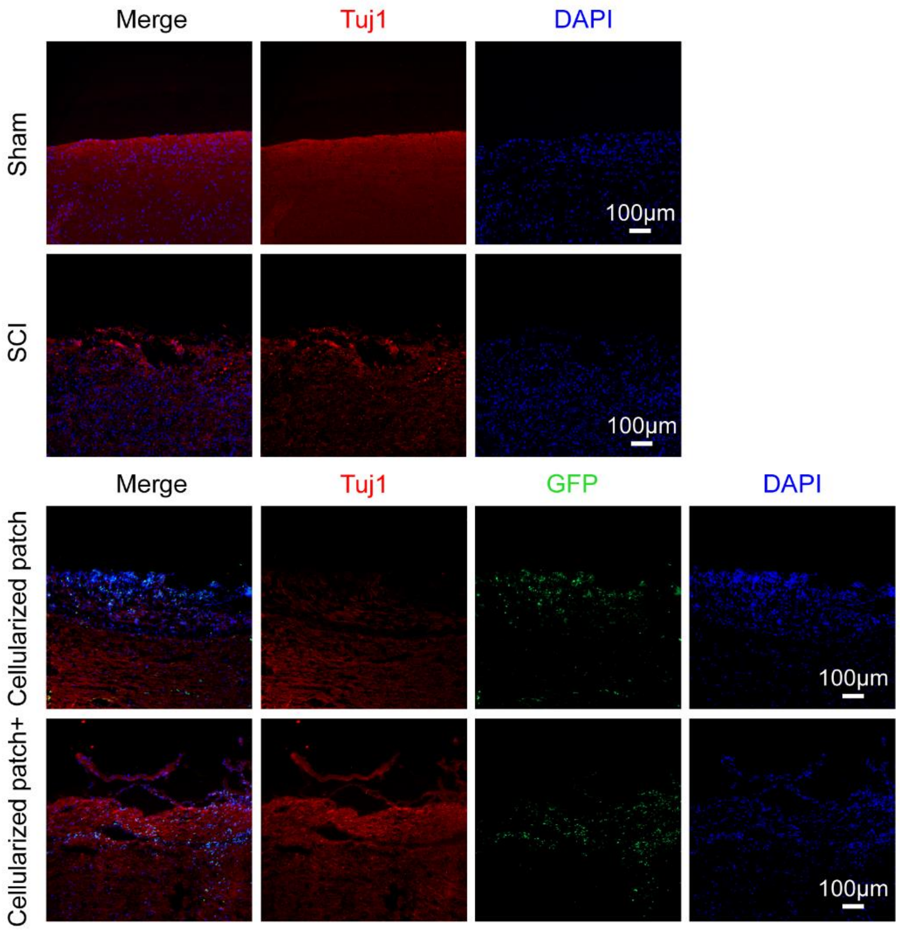

Figure S18. Immunofluorescence images of the spinal cord tissues of the mice in sham, SCI, cellularized patch, and cellularized patch+ groups at day 14. GFP-labeled cells were shown in green, while Tuj1 and nuclei were stained into red and blue, respectively.

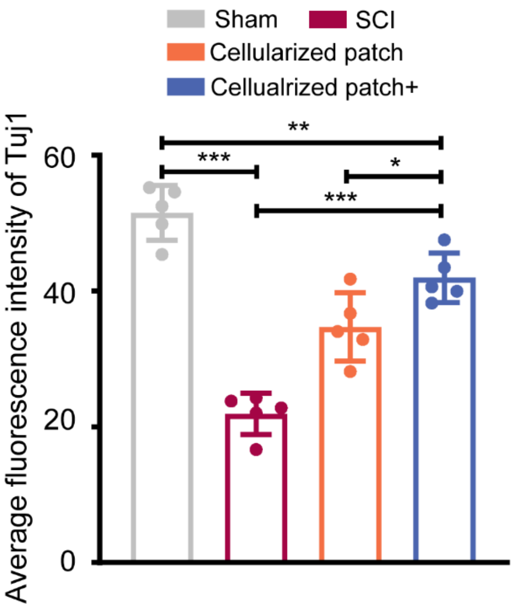

Figure S19. Statistical analysis of the fluorescence intensities of Tuj1 at day 14 based on the immunostaining results in Figure S18. The data were expressed as the mean ± standard deviation (n=5), as analyzed by a one-way ANOVA, with Tukey's multiple comparisons test (*p< 0.05, **p<0.01, and ***p<0.001).

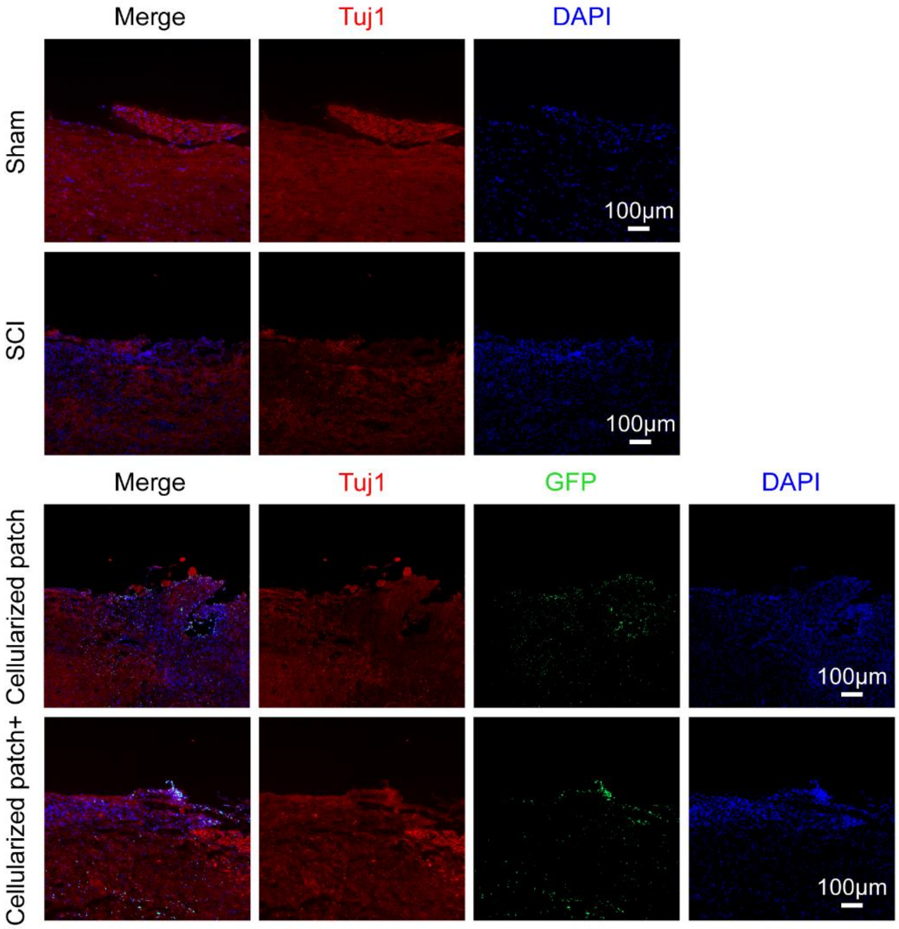

Figure S20. Immunofluorescence images of the spinal cord tissues of the mice in sham, SCI, cellularized patch, and cellularized patch+ groups at day 28. GFP-labeled cells were shown in green, while Tuj1 and nuclei were stained into red and blue, respectively.

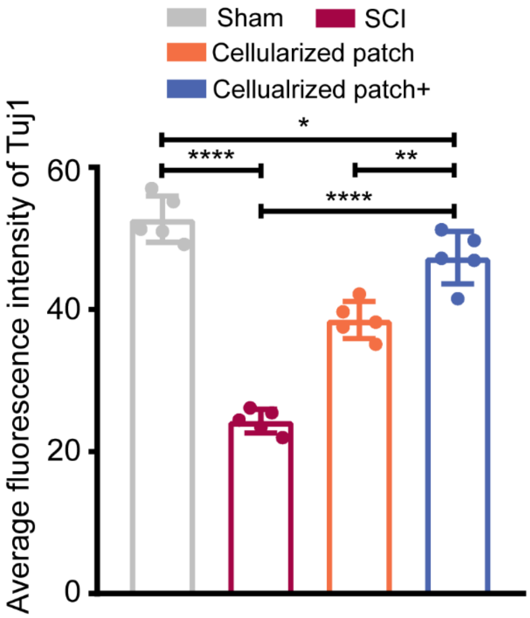

Figure S21. Statistical analysis of the fluorescence intensities of Tuj1 at day 28 based on the immunostaining results in Figure S20. The data were expressed as the mean ± standard deviation (n=5), as analyzed by a one-way ANOVA, with Tukey's multiple comparisons test (*p< 0.05, **p<0.01, and ****p<0.0001).

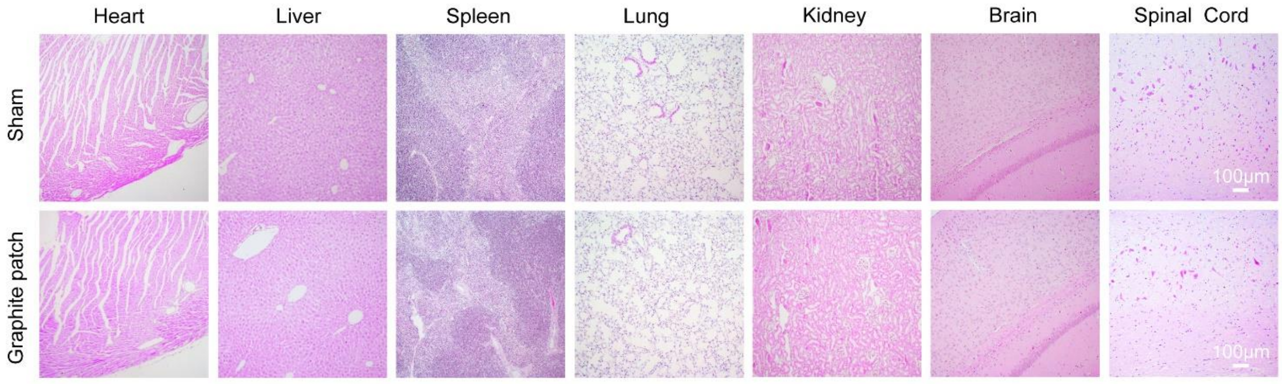

Figure S22. H&E staining of heart, liver, spleen, lung, kidney, brain, and spinal cord of the mice in sham and graphite patch groups at day 28.

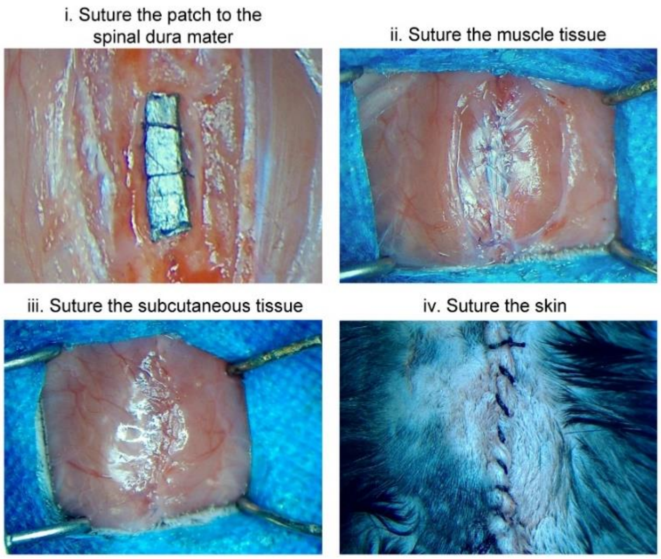

Figure S23. Digital pictures recorded during implantation of the patch: i) suture the patch to the spinal dura mater, ii) suture the muscle tissue, iii) suture the subcutaneous tissue, and iv) suture the skin.

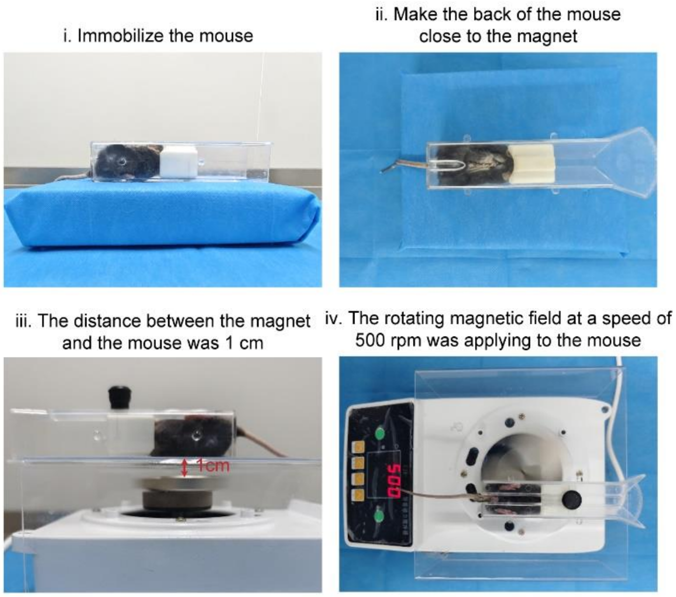

Figure S24. Digital pictures during applying a rotating magnetic field to a mouse: i) immobilize the mouse, ii) making the back of the mouse close to the magnet, iii) the distance between the magnet and the mouse was 1 cm, and iv) the rotating magnetic field at a speed of 500 rpm was applying to the mouse.

Table S1. The identifiers and the type of antibody.

Reagent type	Designation	Source	Identifiers
Primary antibody	Tuj1 Mouse monoclonal	Abcam	ab78078
Primary antibody	MAP2 Mouse monoclonal	Abcam	ab11267
Primary antibody	GFAP Rabbit polyclonal	Abcam	ab7260
Primary antibody	MBP Rabbit monoclonal	Abcam	ab218011
Secondary antibody	Goat anti-rabbit IgG H&L Alexa Fluor 488	Abcam	ab150077
Secondary antibody	Goat anti-mouse IgG H&L Alexa Fluor 594	Abcam	ab150116
Secondary antibody	Goat anti-mouse IgG H&L Alexa Fluor 488	Abcam	ab150113
Secondary antibody	Goat anti-rabbit IgG H&L Alexa Fluor 594	Abcam	ab150080

Table S2. Sequences of RT-qPCR primers.

Gene	Forward primers (5‘-3‘)	Reverse primers (5‘-3‘)
Actin	CGCTGTATTCCCCTCCATCG	CCAGTTGGTAACAATGCCATGT

Tuj1	TATGAAGATGATGACGAGGAATCG	TACAGAGGTGGCTAAAATGGGG
MAP2	TGGAGGAAGCAGCAAGTG	AGGGAGGATGGAGGAAGG
GFAP	CCAAGCCAAACACGAAGCTAA	CATTTGCCGCTCTAGGGACTC
Nestin	TGCCCTAGAGACGGTGTCTCA	AATCGCTTGACCTTCCTCCC
